# Label-Free Comparative Proteomics of Differentially Expressed *Mycobacterium tuberculosis* Protein in Rifampicin-Related Drug-Resistant Strains

**DOI:** 10.3390/pathogens10050607

**Published:** 2021-05-15

**Authors:** Nadeem Ullah, Ling Hao, Jo-Lewis Banga Ndzouboukou, Shiyun Chen, Yaqi Wu, Longmeng Li, Eman Borham Mohamed, Yangbo Hu, Xionglin Fan

**Affiliations:** 1 Department of Pathogen Biology, School of Basic Medicine, Tongji Medical College, Huazhong University of Science and Technology, Wuhan 430030, China; nadeemullah2016@hotmail.com (N.U.); d201781128@hust.edu.cn (L.H.); bangas19@yahoo.fr (J.-L.B.N.); yaqiwu@ustc.edu.cn (Y.W.); d201881254@hust.edu.cn (L.L.); emanborham831@gmail.com (E.B.M.); 2 CAS Key Laboratory of Special Pathogens and Biosafety, Institute of Virology, Chinese Academy of Sciences, Wuhan 430071, China; shiyunchen956@gmail.com

**Keywords:** rifampicin, proteomics, label-free, *Mycobacterium tuberculosis*, drug resistance

## Abstract

Rifampicin (RIF) is one of the most important first-line anti-tuberculosis (TB) drugs, and more than 90% of RIF-resistant (RR) *Mycobacterium tuberculosis* clinical isolates belong to multidrug-resistant (MDR) and extensively drug-resistant (XDR) TB. In order to identify specific candidate target proteins as diagnostic markers or drug targets, differential protein expression between drug-sensitive (DS) and drug-resistant (DR) strains remains to be investigated. In the present study, a label-free, quantitative proteomics technique was performed to compare the proteome of DS, RR, MDR, and XDR clinical strains. We found iniC, Rv2141c, folB, and Rv2561 were up-regulated in both RR and MDR strains, while fadE9, espB, espL, esxK, and Rv3175 were down-regulated in the three DR strains when compared to the DS strain. In addition, lprF, mce2R, mce2B, and Rv2627c were specifically expressed in the three DR strains, and 41 proteins were not detected in the DS strain. Functional category showed that these differentially expressed proteins were mainly involved in the cell wall and cell processes. When compared to the RR strain, Rv2272, smtB, lpqB, icd1, and folK were up-regulated, while esxK, PPE19, Rv1534, rpmI, ureA, tpx, mpt64, frr, Rv3678c, esxB, esxA, and espL were down-regulated in both MDR and XDR strains. Additionally, nrp, PPE3, mntH, Rv1188, Rv1473, nadB, PPE36, and sseA were specifically expressed in both MDR and XDR strains, whereas 292 proteins were not identified when compared to the RR strain. When compared between MDR and XDR strains, 52 proteins were up-regulated, while 45 proteins were down-regulated in the XDR strain. 316 proteins were especially expressed in the XDR strain, while 92 proteins were especially detected in the MDR strain. Protein interaction networks further revealed the mechanism of their involvement in virulence and drug resistance. Therefore, these differentially expressed proteins are of great significance for exploring effective control strategies of DR-TB.

## 1. Introduction

Tuberculosis (TB) remains one of the deadliest infectious diseases worldwide, resulting in 10 million new cases and 1.45 million deaths each year [1]. Currently available anti-TB drugs and vaccines have proved insufficient to contain this scourge. Moreover, the emergence and amplification of multidrug-resistant (MDR) and extensively drug-resistant (XDR) *Mycobacterium tuberculosis* strains have brought serious trouble to current TB prevention and treatment. Early diagnosis of drug-resistant TB (DR-TB) is conducive to timely adjustment of drug regimens for effective cure of TB, thus blocking the spread of DR-TB. Therefore, there is an urgent need to identify candidate targets that might be used for designing early, rapid, and sensitive DR-TB diagnostic and monitoring techniques [2]. 

Proteomic studies provide a novel implementable strategy to combat drug resistance and present a direct way to describe and identify novel target proteins involved in drug resistance directly or indirectly [3]. MDR-TB is TB-resistant to both rifampicin (RIF) and isoniazid (INH), the two vital drugs of the first-line anti-TB agents [4,5]. INH is critically significant after stimulation acts to prevent mycolic acid synthesis and mycobacterial cell wall formation [6]. RIF binds to β subunit of DNA-dependent RNA polymerase and interferes with RNA transcription and elongation. The drug-resistance mechanism of various anti-TB drugs is mainly attributed to specific mutations in the target genes [7,8]. Some genes such as *rpoB* and *rpoC* are currently reported to be related to RIF resistance, while *katG*, *ahpC*, *fabG1*, *kasA*, *ndh,* and *inhA* are noted for INH resistance [9,10]. However, no genes have been found to cause cross-resistance between both RIF and INH. RIF-resistant (RR) strains have increased through their widespread use. Due to its similar resistance to other drugs, especially INH, the RR strain may be considered an alternative MDR-TB marker [11,12]. Currently, diagnostic methods used in hospitals are Drug Susceptibility Testing (DST), Gene Xpert  MTB/RIF assay, and Hain test [13]. However, the problem with current diagnostic methods used in a hospital is the slow growth of *M.* *tuberculosis*, and underlying potential biological safety threats remain insufficient [14]. Xpert MTB/RIF only detects RIF resistance, unable to identify MDR-TB in its earliest form, such as INH mono-resistance. In addition, it must regularly be used together with other tests, i.e., DST, to check and identify each *M.* *tuberculosis* isolate from the whole resistance phenotype [15,16]. In past decades, modern omics methods such as proteomics have had broad application prospects in finding new drug targets due to their complex bacteriological and biochemical characteristics. Proteomics approaches are progressively popular for addressing large-scale and small-scale hypotheses associated with microbial pathogens [2,17]. They constitute an efficient method for determining possible targets for drug development, biomarker discovery, identifying proteins, and drug resistance of pathogens [18].

Previous studies have shown that various chemical labelling techniques lead to limited complementary quantitative information among identified proteins [19]. The label-free quantification (LFQ) approach is mostly based on molecular biophysical properties without conjugated labels, which can avoid false positives and offer more reliable and repeatable test outcomes [20]. In order to sufficiently characterize biomarkers for various DR-TB severity conditions and screen distinct proteins in different stages of TB, the LFQ proteomics technique was used to compare the proteome of drug-sensitive (DS), RIF-resistant (RR), MDR, and XDR strains.

## 2. Results

### 2.1. Up and Down-Regulated Proteins in the RR, MDR, and XDR vs. DS Strains

**Figure 1:**
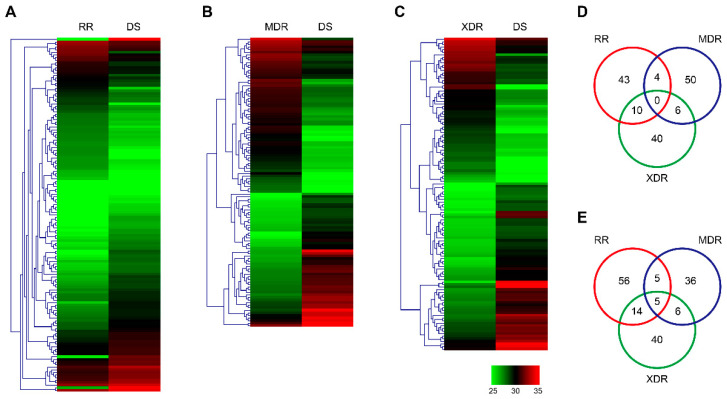
Up and down-regulated proteins between DS and DR strains. (**A**–**C**) Hierarchical clustering of proteins differentially expressed in the RR vs. DS strains (**A**), the MDR vs. DS strains (**B**), and the XDR vs. DS strains (**C**). Log2 ratios were calculated based on LFQ intensity. (**D**,**E**) Venn diagram showed the number of up-regulated (**D**) or down-regulated (**E**) proteins in any of the RR, MDR, XDR strains, when compared to the DS strain. RR, rifampin-resistant; MDR, multidrug-resistant; XDR, extensively drug-resistant; DS, drug-sensitive; LFQ, label-free quantitation.

A total of 2515 proteins were identified through a LFQ technique in DS, RR, MDR, and XDR strains. Heatmaps were generated to visualize the expressions and the clusters of the up-regulated or down-regulated proteins in the RR, MDR, XDR, and DS strains based on their log2 ratios of LFQ intensity (*p* < 0.05). When compared to the DS strain, 58 proteins were up-regulated, while 82 proteins were down-regulated in the RR strain (Figure 1A). Sixty one proteins were significantly increased, while 52 proteins were decreased in the MDR strain (Figure 1B). In addition, 56 proteins were found to be expressed at elevated levels, while 66 proteins showed lower expression in the XDR strain (Figure 1C).

Importantly, both RR and MDR strains showed a high expression of 4 proteins: iniC, Rv2141c, folB, and Rv2561 (Figure 1D). In addition, 10 proteins were increased in the RR and XDR strains, while 6 proteins were increased in the MDR and XDR strains (Figure 1D). However, no protein was found up-regulated in all 3 drug-resistant strains. Functional profiles of the overlapped up-regulated genes were shown in Table 1. The majority of them were involved in intermediary metabolism and respiration. Additionally, 5, 14, and 6 proteins were decreased in the RR & MDR, RR & XDR, and MDR & XDR strains, respectively (Figure 1E). More importantly, the RR, MDR, and XDR strains all showed low expression of 5 proteins: fadE9, espB, espL, esxK, and Rv3175 (Figure 1E). Among them, espB, espL, and esxK were involved in cell wall and cell processes. fadE9 and Rv3175 were involved in lipid metabolism and intermediary metabolism and respiration, respectively (Table 2).

### 2.2. Specifically Expressed and Not Detected Proteins in the RR, MDR, XDR, and DS Strains

**Figure 2:**
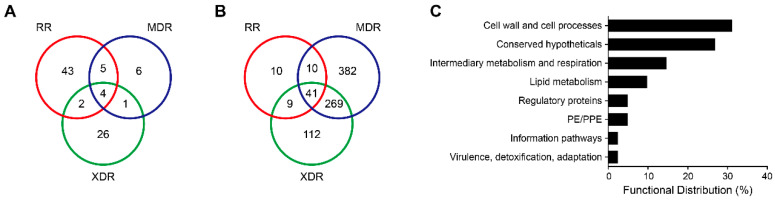
Specifically expressed and not detected proteins between DS and DR strains. (**A**,**B**) Venn diagrams showed the number of proteins specifically expressed (**A**) and not detected (**B**) in any of the RR, MDR, and XDR strains. (**C**) Functional distribution of the 41 not detected proteins in all 3 DR strains according to the TubercuList Functional Category (http://svitsrv8.epfl.ch/tuberculist/ accessed on 18 February 2021). RR, rifampin-resistant; MDR, multidrug-resistant; XDR, extensively drug-resistant; DS, drug-sensitive; PE, proline-glutamic acid; and PPE, proline-proline-glutamic acid.

A total of 87 proteins were identified specifically expressed in any DR strains compared to the DS strain. Among them, 54 were specifically expressed in the RR strain, 16 in the MDR strain, and 33 in the XDR strain (Figure 2A). Importantly, 5 proteins Rv2630, mce2C, moaX, truA, and Rv0200, were especially expressed in the RR and MDR strains. Two proteins, Rv1363c and Rv1360, were especially expressed in the RR and XDR strains. NadB was especially expressed in the MDR and XDR strains. More importantly, 4 proteins were specifically expressed in all 3 DR strains: lprF, mce2R, mce2B, and Rv2627c. Among them, lprF was involved in the cell wall and cell processes, while mce2B was involved in host cell invasion. In addition, mce2R and Rv2627c were regulatory and conserved proteins, respectively (Table 3). 

In addition, 833 proteins were not detected in the DR strains when compared to the DS strain. Among them, 70 proteins were not identified in the RR strain, 702 in the MDR strain, and 431 in the XDR strain (Figure 2B). Importantly, 10, 9, and 269 proteins were not observed in the RR and MDR strains, the RR and XDR strains, and the MDR and XDR strains, respectively. More importantly, all 3 DR strains did not express 41 proteins. The majority of them were involved in cell wall and cell processes (Figure 2C). Functional classification of these undetected proteins in all 3 DR strains is shown in (Table 4).

### 2.3. Differentially Expressed Proteins in the DR Strains

**Figure 3:**
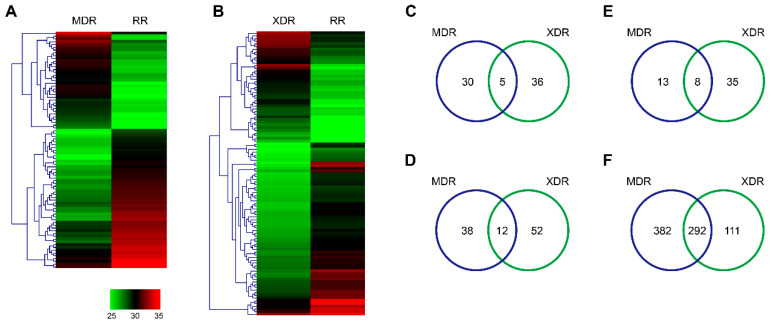
Differentially expressed proteins in the MDR, XDR, and RR strains. (**A**,**B**) Hierarchical clustering of proteins differentially expressed in the MDR vs. RR strains (**A**) and the XDR vs. RR strains (**B**). Log2 ratios were calculated based on LFQ intensity. (**C**–**F**) Venn diagram showed the number of up-regulated (**C**), down-regulated (**D**), especially expressed (**E**), and unexpressed (**F**) proteins in either MDR or XDR strains, when compared to the RR strain. RR, rifampin-resistant; MDR, multidrug-resistant; XDR, extensively drug-resistant; LFQ, label-free quantitation.

We further compared the differentially expressed proteins in the 3 DR strains. Compared to the RR strain, 35 proteins were up-regulated, while 50 proteins were down-regulated in the MDR strain (Figure 3A). Forty-one proteins were significantly increased, while 64 proteins were decreased in the XDR strain (Figure 3B). Importantly, both MDR and XDR strains showed high expression of 5 proteins: Rv2272, smtB, lpqB, icd1, and folK (Figure 3C, Table 5) and low expression of 12 proteins: esxK, PPE19, Rv1534, rpmI, ureA, tpx, mpt64, frr, Rv3678c, esxB, esxA, and espL (Figure 3D, Table 6). In addition, 22 and 44 proteins were specifically expressed in the MDR and XDR strains, respectively, when compared to the RR strain (Figure 3E). Importantly, 8 proteins were specifically expressed in the MDR and XDR strains: nrp, PPE3, mntH, Rv1188, Rv1473, nadB, PPE36, and sseA (Figure 3E, Table 7). In contrast, a total of 785 proteins were not detected in either MDR or XDR strains when compared to the DS strain (Figure 3F). Among them, 292 proteins were not identified in both MDR and XDR strains (Figure 3F), most of which were conserved hypotheticals (Figure S1).

**Figure 4:**
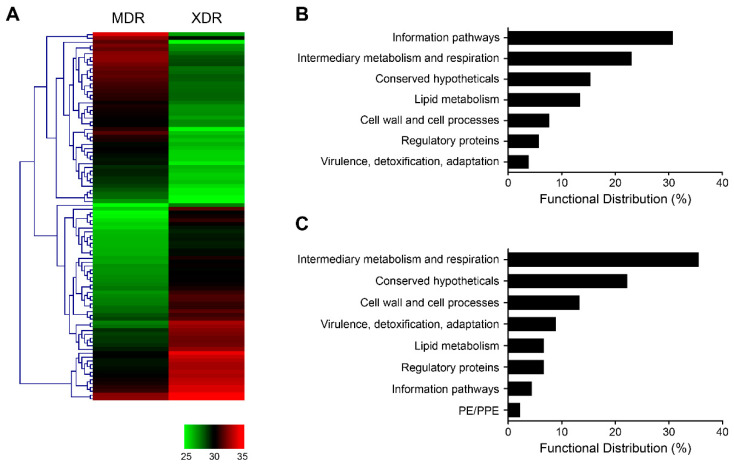
Up and down-regulated proteins between MDR and XDR strains. (**A**) Hierarchical clustering of proteins differentially expressed in the XDR vs. MDR strains. Log2 ratios were calculated based on LFQ intensity. (**B**,**C**) Functional distribution of the up-regulated (**B**) or down-regulated (**C**) proteins in the XDR strain, when compared to the MDR strain, according to the TubercuList Functional Category (http://svitsrv8.epfl.ch/tuberculist/ accessed on 18 February 2021). RR, rifampin-resistant; MDR, multidrug-resistant; XDR, extensively drug-resistant; LFQ, label-free quantitation.

In addition, 52 proteins were identified up-regulated, while 45 proteins were down-regulated in the XDR strain when compared to the MDR strain (Figure 4A). The majority of the up-regulated proteins in the XDR strain were involved in information pathways (Figure 4B), while the down-regulated proteins were involved in intermediary metabolism and respiration (Figure 4C). Moreover, a total of 316 proteins were especially expressed in the XDR strain, while 92 proteins were especially expressed in the MDR strain (Figure S2). Most of the especially expressed proteins in either the XDR or MDR strain belonged to intermediary metabolism and respiration, conserved hypotheticals, and cell wall and cell processes.

### 2.4. The Molecular Mechanisms of the Differentially Expressed Proteins in the DR vs. DS Strains

To better understand the association of the 20 up-regulated proteins in DR strains, we mapped them onto STRING database to build a PPI network (Figure S3A). Only Rv1780 could not play a part in the construction of the network. Function enrichment analysis against gene ontology (GO) in this network showed that for biological processes, this network was enriched in organonitrogen compound metabolic process, water-soluble vitamin biosynthetic process, riboflavin biosynthetic and metabolic process, drug metabolic process, and folic-acid-containing compound biosynthetic and metabolic process. For molecular function, it was significantly enriched in proteasome binding, purine ribonucleotide binding, and drug binding. For cellular components, it was significantly enriched in the proteasome complex, cytoplasm, and cell wall (Tables S2–S4).

Thirty down-regulated proteins of DR strains were mapped onto STRING database to build a PPI network (Figure S3B). PPE36, rpmB1, and Rv3295 could not play roles in the construction of the network. Function enrichment analysis against GO in this network showed that for biological processes, this network was enriched in pathogenesis, cellular oxidant detoxification, protein transmembrane transport, evasion or tolerance of host immune response, response to an external stimulus, and cellular response to a chemical stimulus. For molecular function, it was significantly enriched in antioxidant activity, peroxidase activity, and peroxiredoxin activity. For cellular components, it was significantly enriched in the extracellular region, cell wall, and plasma membrane (Tables S5–S7).

Other 12 specific expressed proteins of DR strains were mapped onto STRING database to build a PPI network (Figure S3C). Only Rv1360 could not take part in the construction of the network. Function enrichment analysis against GO in this network showed that for biological processes, this network was enriched in coenzyme biosynthetic process, NAD biosynthetic and metabolic process, and Mo-molybdopterin cofactor metabolic and biosynthetic process. For cellular components, it was significantly enriched in the molybdopterin synthase complex (Tables S8 and S9).

In addition, 41 undetected proteins in the DR strains were mapped onto STRING database to build a PPI network (Figure S3D). Only Rv1424c could not participate in the construction of the network. Function enrichment analysis against GO in this network showed that for biological processes, this network was enriched in peptidoglycan-based cell wall biogenesis and antibiotic transport. For cellular components, it was significantly enriched in polyketide synthase complex and catalytic complex (Tables S10 and S11).

## 3. Discussion

Due to the emergence of MDR and XDR strains, current vaccination strategies and anti-TB drugs have been proved insufficient to contain this considerable trouble [5]. Anti-TB drug resistance has been reported to be associated with specific mutations in the regulatory and/or structural regions of target genes, as mentioned above [5,21]. In the present study, we identified 2515 proteins through LFQ among DS, RR, MDR, and XDR strains. We found iniC, Rv2141c, folB, and Rv2561 were up-regulated in both RR and MDR strains, while fadE9, espB, espL, esxK, and Rv3175 were down-regulated in the three DR strains when compared to the DS strain. In addition, lprF, mce2R, mce2B, and Rv2627c were specifically expressed in the three DR strains, and 41 proteins were not detected compared to the DS strain. Functional category showed that these differentially expressed proteins were mainly involved in the cell wall and cell processes. Compared to the RR strain, Rv2272, smtB, lpqB, icd1, and folK were up-regulated, while esxK, PPE19, Rv1534, rpmI, ureA, tpx, mpt64, frr, Rv3678c, esxB, esxA, and espL were down-regulated in both MDR and XDR strains. Additionally, nrp, PPE3, mntH, Rv1188, Rv1473, nadB, PPE36, and sseA were specifically expressed in both MDR and XDR strains, whereas 292 proteins were undetected when compared to the RR strain. When compared between MDR and XDR strains, 52 proteins were identified up-regulated, while 45 proteins were down-regulated in the XDR strain. Three hundred and sixteen proteins were specially expressed in the XDR strain, while 92 proteins were specially expressed in the MDR strain. Bioinformatics studies like protein–protein interactions showed their participation in virulence and drug resistance [22]. Therefore, these differentially expressed proteins are of great significance for exploring effective control strategies of DR-TB.

Proteomics play significant roles in the medical research era, for example, for understanding the relation between proteins, discovery of drugs, and diagnosis of diseases. In our report, we found that iniC, Rv2141c, folB, and Rv2561 were up-regulated in both RR and MDR strains compared to the DS strains. IniC (Rv0343) is INH inducible protein, with the highest tendency triggering resistance for both INH and EMB [23]. The gene iniC was structured in an operon, controlled by iniBAC promoter, and up-regulated via several cell envelope inhibitors such as INH and other cell wall-active drugs [24]. Rv2141c is a conserved protein, which is also expressed in *Mycobacterium smegmatis* [25] and significantly inhibits cell death [26]. FolB (Rv3607c) is an metabolic persister gene involved in folate/dihydroneopterin biosynthetic pathway. FolB protein is essential to the survival of *M. tuberculosis* and might be used as novel targets for the development of drugs [27]. Rv2561 is a “conserved hypothetical” protein with unknown functions, up-regulated in broth-grown *M. tuberculosis* H37Ra and H37Rv strains [28]. Rv2561 was related to druggable proteins [29]. We demonstrated the corresponding PPI network of the up-regulated proteins of DR strains was enriched in many metabolic pathways. Metabolic pathways play a significant role in drug resistance as part of the global response and a novel strategy against DR bacteria [30]. Potential drug targets from these related pathways include amino acid metabolism, energy metabolism, lipid metabolism, and vitamin cofactor biosynthetic pathways. These pathways could be beneficial for the discovery of broad-spectrum drugs [31]. Several folate pathway determinates have been attributed to the sulfonamide resistance in *M. tuberculosis*, and the frontline anti-TB drugs such as ETH, INH, and PAS have been revealed as potential inhibitors of the folate biosynthetic pathway [32].

We carried out a proteomics study of *M. tuberculosis* differentially expressed proteins and determined that espB, espL, esxK, and Rv3175 were down-regulated in the three DR strains when compared to the DS strain. EspB (Rv3881c) protein is a substrate for the ESX-1 type VII protein secretion system, which is required for the virulence and growth in macrophages, [33]. EspL (Rv3880c) interacts with EspD and has been discovered to maintain the stability of the effector proteins and ESX-1 substrates that are involved in ESAT-6 secretion and stabilization of EspE expression. EspL has a strong DNA-binding ability and plays a role as a chaperone [34]. EsxK (Rv1197) is a putative interacting protein that belongs to primary secretory (ESX-6) system antigenic targets [35]. Rv3175 is a possible amidase involved in cellular metabolism [36]. However, this basal level of amidase activities is sufficient for the utilization of nicotinamide by *Mycobacterium bovis*, whereas it is not enough to regulate PZA susceptibility [37]. We found the corresponding PPI network of the down-regulated proteins of DR strains was enriched in antioxidant activity, peroxidase activity, and peroxiredoxin activity. The reduced activity of oxidative stress in *M. tuberculosis* is crucial for resistance to clofazimine, a peroxide-generating drug [38]. The decrease or loss of peroxidase activity is the most common genetic change related to INH resistance [38].

In addition, lprF, mce2R, mce2B, and Rv2627c were specifically expressed in the three DR strains when compared to the DS strains. LprF (Rv1368) modifications in various strains resulted from resisting antimicrobial agents and conserved lipoprotein synthesis pathway. Lipoproteins are virulence factors of *M. tuberculosis*; *M. bovis* is considered as a model organism to study the mutation of lipoprotein in Mycobacteria [39]. LprF possess transmembrane helices and were found to be potential candidates for serodiagnostic techniques [39,40]. Similarly, lprF crystal structure has been determined, which is a functional homolog to lprG that offers lprF transferring diacylated glycolipid from the plasma membrane to the cell wall [41]. Mce2R (Rv0586) is a Guntur/FadR family protein in *M. tuberculosis* identified as FadR homolog that acts as a fatty acid reactive transcriptional regulator of *M. tuberculosis* mycolic acid biosynthesis [42]. Protein DNA interaction (PDI) data demonstrate that novel target genes (mce2R) play essential roles in the regulation of multidrug resistance, cell wall synthesis, and intracellular growth of *M. tuberculosis* [43]. Mce2B (Rv0590) is mce family protein thought to be involved in host cell invasion [44]. However, mce operons have been associated with the attenuation of *M. tuberculosis* virulence [45]. Rv2627c belongs to DosR regulon encoded proteins and latency-associated antigens in case of latently infected individuals, which induced high levels and long-term of IFN-γ responses [46,47]. Other 41 proteins were not detected in the three DR strains when compared to the DS strains, and several such as Rv3321c (VapB44), possible antitoxin protein [48], Rv3426 (PPE58), PPE family protein, and conserved membrane proteins Rv2219A [49] and Rv3760 remain unknown function. They might be used as a potential drug target for future research.

Moreover, we further analyzed the differentially expressed proteins among the 3 DR strains and found Rv2272, smtB, lpqB, icd1, and folK were highly expressed in both MDR and XDR strains when compared to the RR strain. Rv2272 is a transmembrane protein involved in Sigma factor E regulation during macrophage infection, which is helpful for the pathogen to deal with environmental stress and to inhibit the host antibacterial responses [50]. SmtB (Rv2358) is a Zinc-binding transcriptional regulator that belongs to the ArsR family protein, which affects the elasticity of the N-terminal trans-activation domain and shows amino acid variations specific to the Beijing lineage of *Mtb* [51]. LpqB (Rv3244c) is a putative lipoprotein, which plays an important role in cell growth and cell wall metabolism [52]. The encoding gene of lpqB is located immediately downstream of MtrA and MtrB, so that lpqB might act as a modulator of MtrB activity [53]. Icd-1 (Rv3339c) has been identified in the *M. tuberculosis* genome as a probable isocitrate dehydrogenase gene encoded for functional energy cycle enzymes [54]. Folk (Rv3606c) protein is involved in the dihydrofolate biosynthesis pathway, validated as a drug target [55,56].

On the other hand, 12 proteins, namely, esxK, PPE19, Rv1534, rpmI, ureA, tpx, mpt64, frr, Rv3678c, esxB, esxA, and espL, were down-regulated in both MDR and XDR strains when compared to the RR strain. Except for esxK and espL discussed above, PPE19 (Rv1361c) has been identified as a putative target of MtrA [57], and a genomics indicator of susceptibility to amoxicillin-clavulanate [58]. Rv1534 protein is possibly involved in a transcriptional process, metabolism, and cell development [59]. RpmI (Rv1642) is an active virulence operon involved in protein synthesis and is associated with invasion and intercellular resistance [60]. UreA (Rv1848) is involved in the conversion of urea to ammonia (NH3). Urease, encoded within an operon consists of ureA subunit, is important for the survival of *M. tuberculosis* in the lung tissue and remains an attractive target for chemotherapeutic TB [61]. TpX (Rv1932) thiol peroxidase has been characterized as a cysteine peroxiredoxin which serves as a reaction site for either oxidizing or reducing substrates [62]. It is the most efficient enzyme to protect *M. tuberculosis* against oxidative and nitrosative stress and is overexpressed in the presence of ofloxacin (OFX) and moxifloxacin (MOX) [63,64]. Mpt64 (Rv1980c) binds to streptomycin, whose overexpression might cumulatively neutralize or compensate for the drug’s effect and contribute to streptomycin resistance [65]. Frr (Rv2882c) encodes the ribosome recycling factor and maintains translation fidelity by preventing aberrant reinitiation of translation downstream of the mRNA stop codon [66]. Frr protein might be involved in the ofloxacin modulation or neutralization and causes ofloxacin resistance [67]. Rv3678c conserved protein has an unknown function, studied as a regulator against stress-responsive gene [68]. EsxB (Rv3874) and EsxA (Rv3875) are two major substrates of the ESX-1 system. EsxA helps breach the phagosomal membrane and facilitate the escape of *M. tuberculosis* into the cytosol [69,70].

More importantly, both MDR and XDR strains specifically expressed 8 proteins: nrp, PPE3, mntH, Rv1188, Rv1473, nadB, PPE36, and sseA, when compared to the RR strain. Nrp (Rv0101) is a peptide synthetase that plays a role in in vivo infection during survival [58]. PPE3 (Rv0280) and PPE36 (Rv2108) are members of the PPE family with an unknown function. PPE3 was studied as a putative genomic marker of amoxicillin/clavulanate susceptibility [58], while PPE36 was involved in the heme utilization by *M. tuberculosis* [71]. MntH (Rv0924c) is an integral membrane protein of *M. tuberculosis* that mediates divalent cation transportation [72]. Rv1188 gene encodes for proline dehydrogenase. Proline metabolism is an important player in the persistence of *M. tuberculosis* through detoxification of methyl-glyoxal [73]. Rv1473 is a predicted drug efflux pump involved in mycobacteria intrinsic drug resistance through the efflux process [74]. NadB (Rv1595) is an L-aspartate oxidase involved in the differentially regulated conversion of aspartate to NAD^+^ in response to nitrogen stress [49]. SseA (Rv3283) is a thiosulfate sulfurtransferase down-regulated in the Beijing B0/W148 strains, leading to enhanced oxidative DNA damage, mutations, and drug resistance [75].

Therefore, our comparative proteomics study obtained differentially expressed candidate proteins of DR strains, and the functional category of these proteins laid the infrastructure for the establishment of effective control strategies for DR-TB.

## 4. Materials and Methods 

### 4.1. Mycobacterial Growth

Three kinds of clinical *Mtb* DR strains, namely, RR, MDR, and XDR strains, were obtained from the DR-TB patients in Wuhan Pulmonary Hospital (Wuhan, China). The standard H37Rv strain was regarded as the DS strain. All strains were grown in Middlebrook 7H9 liquid medium (Difco Laboratories, Sparks, MD, USA) with Tween 80 0.05% and ADC 10% enrichment containing bovine serum albumin (BSA)-dextrose-catalase 0.5% (Difco) at 37 °C for 10–14 days to their mid-log phase. For proteomic analysis, strains were washed, passaged one time in Sauton’s medium, and then grown for 2 days to early log phase. 

### 4.2. DST of Clinical DR Strains

All experiments using live clinical *Mtb* strains were conducted in the biosafety level 2 plus laboratory of Wuhan Pulmonary Hospital. All strains were tested for drug susceptibility against 6 anti-TB drugs according to the World Health Organization (WHO) and Clinical and Laboratory Quality Institute (CLSI) guidelines. Löwenstein-Jensen commercial media (Wuhan, China) containing antibiotics were used in the proportion methods to test all antibiotics. The critical concentrations for INH (Sigma, Oakville, ON, Canada) were 0.2 mg/L, RIF (Sigma) 40.0 mg/L, ethambutol (EMB, Sigma) 2.0 mg/L, levofloxacin (LFX, Sigma) 2.0 mg/L, moxifloxacin (MOX, Sigma) 2.0 mg/L, and kanamycin (KAN, Sigma) 30.0 mg/L, respectively. After 3 weeks of incubation at 37 °C, their DST results were confirmed (Table S1). 

### 4.3. Whole Cell Lysate Protein Extraction of Mycobacteria

The mycobacterial lysate was obtained under ultrasonic decomposition at an interval of 10 s (100 W, 10 circles). Then, samples were placed in a bath full of boiling water for 10 min and clarified by centrifugation (13,400 rpm for 30 min). The supernatant containing the whole cell lysate proteins was retained and determined the concentrations with BCA according to the manufacturer’s instructions. The lysate was sterilized through a filter membrane with the pore diameter of 0.22 μm (Pall Corporation, Ann Arbour, MI, USA) and stored at −80 °C until further analysis [76]. The lysate proteins were identified by 12% SDS-PAGE.

### 4.4. Filter-Aided Sample Preparation (FASP) Digestion

FASP digestion was performed following the published protocol [77]. Briefly, 30 μg of each protein extract from different mycobacterial strains was added with dithiothreitol (100 mM) and incubated at 95 °C for 5 min. There were three replicate experiments for each strain. Samples were mixed up with 200 μL of UA (8 M Urea, 150 mM Tris-HCl, pH 8.0) and centrifuged at 14,000× *g* for 15 min at 25 °C. The clean-up step was repeated. Then, samples were added with 100 μL iodoacetamide (IAA) solution (50 mM IAA in UA) and mixed at 600 rpm in a thermo-mixer for 1 min, incubated without mixing for 30 min in the dark, and centrifuged at 14,000× *g* for 10 min. Then, samples were mixed up with 100 μL UA buffer twice, centrifuged at 14,000× *g* for 10 min, and mixed up with 100 μL NH_4_HCO_3_ buffer twice. Then, samples were added with 40 μL Trypsin buffer (2 μg Trypsin in 40 μL NH_4_HCO_3_ buffer) mixed at 600 rpm in a thermo-mixer for 1 min, and incubated for 16–18 h at 37 °C. The filtrate was desalted using C18-SD Extraction Disk Cartridge and estimated by measuring absorbance at 280 nm.

### 4.5. LC-MS/MS Analysis

An EASY-nLC 1000 ultra-high pressure system (Thermo Scientific/Proxeon, Odense, Denmark) was used for LC-MS/MS analysis. Briefly, about 2 μg of enzymatic hydrolysates was applied to a chromatographic column, packed with 3 μm Reprosil pur 120 C18 material, and balanced with mobile phase A (2% acetonitrile, 0.1% formic acid). Samples were separated using a 100 min gradient from 0 to 45%, 8 min from 45 to 100%, and 10 min maintained at 100% of mobile phase B (84% acetonitrile, 0.1% formic acid) at 400 Nl/min. Enzymatic hydrolysates were analyzed using automated data-dependent acquisition on a Q Exactive™ HF hybrid Quadrupole Orbitrap™ mass spectrometer (Thermo Finnigan, Germany). Each MS scan (300–1800 m/z range) was acquired at a resolution of 70,000 and was followed by Top20 MS/MS scans triggered above an intensity of 17,500 using higher-energy C-trap dissociation (HCD).

### 4.6. LFQ Analysis

**Figure 5:**
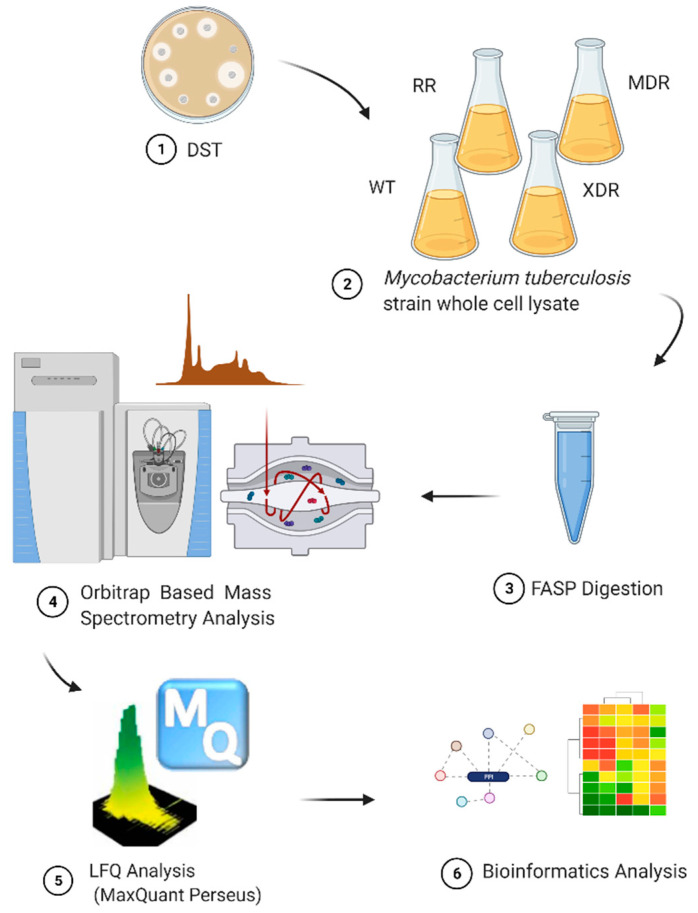
Workflow of the label-free quantitative (LFQ) proteomics.

Raw label-free MS/MS data were processed using Maxquant (version 1.3.0.5) with default parameters. Parameters for protein searching were defined in Uniprot_mycobacterium_tuberculosis_25618_H37RV_6541_20161114.fasta database (updated on 14 November 2016). For statistical evaluation of the data obtained, the posterior error probability and false discovery rate (FDR) were used. The FDR of 0.01 for proteins and peptides was required. The peptide identification was based on a search with an initial mass deviation of precursor ion of up to 20 ppm, with further data recalibrations and a new search with a 6-ppm deviation. MS/MS mass deviation was set to 20 ppm for Q Exactive data and 0.5 Da for any LTQ-Orbitrap data. To match identifications across different replicated and adjacent fractions, the “match between runs” options in MaxQuant was enabled within a time window of 2 min. Proteins and peptides identified and quantified by at least two out of three replicates were used to compare different sample preparation protocols [78]. Significance B test with a FDR value < 5% was applied to identify proteins for which the abundance was significantly changed between the two *Mtb* strains [79]. Workflow of the label-free quantitative (LFQ) proteomics was shown in Figure 5.

### 4.7. Bioinformatics Analysis

Proteomics data were performed by using hierarchical cluster analysis Multi Experiment Viewer (MeV 4.0) software. Tuberculist database was used for functional categories of the differently expressed genes (http://svitsrv8.epfl.ch/tuberculist/ accessed on 18 February 2021). The differently expressed proteins identified from the proteomics results were mapped onto the STRING database (http://string-db.org/) to develop protein–protein interaction (PPI) networks (Confidence score > 0.4, accessed on 18 February 2021). Functional enrichments of each network were analyzed and displayed on the webpage.

## Figures and Tables

**Table 1:** Up-regulated genes in the DR strains compared to the DS strain, sorted in order of gene number.

Locus Tag.	Gene Symbol	Function	Product	Functional Category	Strains
Rv0343	iniC	Unknown	Isoniazid inductible gene protein IniC	cell wall and cell processes	RR and MDR
Rv0408	pta	Involved at the last step (of two) in the conversion of acetate to acetyl-CoA [catalytic activity: acetyl-CoA + phosphate = CoA + acetyl phosphate]	Probable phosphate acetyltransferase Pta (phosphotransacetylase)	intermediary metabolism and respiration	RR and XDR
Rv0593	lprL	Unknown, but thought to be involved in host cell invasion.	Possible Mce-family lipoprotein LprL (Mce-family lipoprotein Mce2E)	cell wall and cell processes	RR and XDR
Rv0652	rplL	Involved in translation mechanisms: seems to be the binding site for several of the factors involved in protein synthesis and appears to be essential for accurate translation.	50S ribosomal protein L7/L12 RplL (SA1)	information pathways	RR and XDR
Rv1185c	fadD21	Function unknown but supposedly involved in lipid degradation.	Probable fatty-acid-AMP ligase FadD21 (fatty-acid-AMP synthetase) (fatty-acid-AMP synthase)	lipid metabolism	RR and XDR
Rv1187	rocA	Involved in the arginase pathway [catalytic activity: 1-pyrroline-5-carboxylate + NAD(+) + H(2)O = l-glutamate + NADH]	Probable pyrroline-5-carboxylate dehydrogenase RocA	intermediary metabolism and respiration	MDR and XDR
Rv1416	ribH	Riboflavin synthase is a bifunctional enzyme complex involved in riboflavin synthesis. Riboflavin synthase catalyzes the formation of riboflavin from 5-amino-6-(1’-d)-ribityl-amino-2,4(1*H*,3*H*)-pyrimidinedione and l-3,4-dihydrohy-2-butanone-4-phosphate via 6,7-dimethyl-8-lumazine. The beta subunit catalyzes the condensation of 5-amino-6-(1’-d)-ribityl-amino-2,4(1*H*,3*H*)-pyrimidinedione with L-3,4-dihydrohy-2-butanone-4-phosphate yielding 6,7-dimethyl-8-lumazine	Probable riboflavin synthase beta chain RibH (6,7-dimethyl-8-ribityllumazine synthase) (DMRL synthase) (lumazine synthase)	intermediary metabolism and respiration	RR and XDR
Rv1493	mutB	Involved in propionic acid fermentation. Catalyzes the isomerization of succinyl-CoA to methylmalonyl-CoA during synthesis of propionate from tricarboxylic acid-cycle intermediates [catalytic activity: (*R*)-2-methyl-3-oxopropanoyl-CoA = succinyl-CoA]	Probable methylmalonyl-CoA mutase large subunit MutB (MCM)	lipid metabolism	RR and XDR
Rv1711	Rv1711	Function unknown	Conserved hypothetical protein	Conserved hypotheticals	RR and XDR
Rv1780	Rv1780	Function unknown	Conserved protein	Conserved hypotheticals	RR and XDR
Rv2115c	mpa	Involved in proteasomal protein degradation	Mycobacterial proteasome ATPase Mpa	cell wall and cell processes	MDR and XDR
Rv2141c	Rv2141c	Unknown	Conserved protein	intermediary metabolism and respiration	RR and MDR
Rv2148c	Rv2148c	Unknown	Conserved protein	Conserved hypotheticals	RR and XDR
Rv2272	Rv2272	Unknown	Probable conserved transmembrane protein	cell wall and cell processes	MDR and XDR
Rv2561	Rv2561	Function unknown	Conserved hypothetical protein	Conserved hypotheticals	RR and MDR
Rv2851c	Rv2851c	Acetylation, substrate unknown	GCN5-related N-acetyltransferase	intermediary metabolism and respiration	RR and XDR
Rv3244c	lpqB	Unknown	Probable conserved lipoprotein LpqB	cell wall and cell processes	MDR and XDR
Rv3371	Rv3371	May be involved in synthesis of triacylglycerol	Possible triacylglycerol synthase (diacylglycerol acyltransferase)	lipid metabolism	MDR and XDR
Rv3606c	folK	Involved in dihydrofolate biosynthesis (at the first step) [catalytic activity: ATP + 2-amino-4-hydroxy-6-hydroxymethyl-7,8-dihydropteridine = AMP + 2-amino-7,8-dihydro-4-hydroxy-6-(diphosphooxymethyl)pteridine]	2-amino-4-hydroxy-6-hydroxymethyldihydropteridine pyrophosphokinase FolK (7,8-dihydro-6-hydroxymethylpterin-pyrophosphokinase) (HPPK) (6-hydroxymethyl-7,8-dihydropterin pyrophosphokinase) (PPPK) (2-amino-4-hydroxy-6-hydroxymethyldihydropteridine diphosphokinase) (7,8-dihydro-6-hydroxymethylpterin-diphosphokinase) (6-hydroxymethyl-7,8-dihydropterin diphosphokinase)	intermediary metabolism and respiration	MDR and XDR
Rv3607c	folB	Involved in folate biosynthesis. Catalyzes the conversion of 7,8-dihydroneopterin to 6-hydroxymethyl-7,8-dihydropterin [catalytic activity: 2-amino-4-hydroxy-6-(d-erythro-1,2,3-trihydroxypropyl)-7,8-dihydropteridine = 2-amino-4-hydroxy-6-hydroxymethyl-7,8-dihydropteridine + glycolaldehyde]	Probable dihydroneopterin aldolase FolB (DHNA)	intermediary metabolism and respiration	RR and MDR

**Table 2:** Down-regulated genes in the DR strains compared to the DS strain, sorted in order of gene number.

Locus Tag	Gene Symbol	Function	Product	Functional Category	Strains
Rv0077c	Rv0077c	Function unknown; probably involved in cellular metabolism.	Probable oxidoreductase	Intermediary metabolism and respiration	RR and XDR
Rv0105c	rpmB	Possibly involved in a translation mechanism	50S ribosomal protein L28-1 RpmB1	Information pathways	RR and MDR
Rv0282	eccA3	Unknown	ESX conserved component EccA3. ESX-3 type VII secretion system protein.	Cell wall and cell processes	RR and XDR
Rv0283	eccB3	Unknown	ESX conserved component EccB3. ESX-3 type VII secretion system protein. Possible membrane protein.	Cell wall and cell processes	RR and XDR
Rv0752c	fadE9	Function unknown, but involved in lipid degradation.	Probable acyl-CoA dehydrogenase FadE9	lipid metabolism	RR, MDR and XDR
Rv0753c	mmsA	Plays a role in valine and pyrimidine metabolism	Probable methylmalonate-semialdehyde dehydrogenase	Intermediary metabolism and respiration	RR and XDR
Rv0969	ctpV	Metal cation-transporting ATPase; possibly catalyzes the transport of an undetermined metal cation with the hydrolysis of ATP	Probable metal cation transporter P-type ATPase CtpV	Cell wall and cell processes	RR and XDR
Rv1196	PPE18	Unknown	PPE family protein PPE18	PE/PPE	MDR and XDR
Rv1197	esxK	Function unknown	ESAT-6 like protein EsxK (ESAT-6 like protein 3)	Cell wall and cell processes	RR, MDR and XDR
Rv1198	esxL	Function unknown	Putative ESAT-6 like protein EsxL (ESAT-6 like protein 4)	Cell wall and cell processes	RR and MDR
Rv1793	esxN	Function unknown	Putative ESAT-6 like protein EsxN (ESAT-6 like protein 5)	Cell wall and cell processes	RR and MDR
Rv1932	tpx	Has antioxidant activity. Could remove peroxides or H(2)O(2)	Probable thiol peroxidase Tpx	Virulence, detoxification, adaptation	MDR and XDR
Rv1980c	mpt64	Unknown	Immunogenic protein Mpt64 (antigen Mpt64/MPB64)	Cell wall and cell processes	MDR and XDR
Rv2094c	tatA	Involved in protein export:	Sec-independent protein translocase membrane-bound protein TatA	Cell wall and cell processes	RR and XDR
Rv2108	PPE36	Unknown	PPE family protein PPE36	PE/PPE	MDR and XDR
Rv2284	lipM	Hydrolysis of lipids (bound ester).	Probable esterase LipM	Intermediary metabolism and respiration	RR and XDR
Rv2428	ahpC	Involved in oxidative stress response	Alkyl hydroperoxide reductase C protein AhpC (alkyl hydroperoxidase C)	Virulence, detoxification, adaptation	RR and MDR
Rv2945c	lppX	Unknown	Probable conserved lipoprotein LppX	Cell wall and cell processes	RR and XDR
Rv3174	Rv3174	Function unknown; involved in cellular metabolism.	Probable short-chain dehydrogenase/reductase	Intermediary metabolism and respiration	RR and XDR
Rv3175	Rv3175	Function unknown; involved in cellular metabolism.	Possible amidase (aminohydrolase)	intermediary metabolism and respiration	RR, MDR and XDR
Rv3275c	purE	Involved in purine biosynthesis (sixth step).	Probable phosphoribosylaminoimidazole carboxylase catalytic subunit PurE (air carboxylase) (AIRC)	Intermediary metabolism and respiration	RR and XDR
Rv3284	Rv3284	Function unknown	Conserved hypothetical protein	Conserved hypotheticals	RR and XDR
Rv3288c	usfY	Function unknown	Putative protein UsfY	Conserved hypotheticals	RR and XDR
Rv3295	Rv3295	Involved in transcriptional mechanism	Probable transcriptional regulatory protein (probably TetR-family)	Regulatory proteins	RR and XDR
Rv3311	Rv3311	Function unknown	Conserved protein	Conserved hypotheticals	RR and XDR
Rv3327	Rv3327	Involved in the transposition in the insertion sequence element IS1547.	Probable transposase fusion protein	Insertion seqs and phages	RR and MDR
Rv3874	esxB	Unknown. Exported protein cotranscribed with Rv3875|MT3989|MTV027.10.	10 kDa culture filtrate antigen EsxB (LHP) (CFP10)	Cell wall and cell processes	MDR and XDR
Rv3875	esxA	Unknown. Elicits high level of inf-gamma from memory effector cells during	6 kDa early secretory antigenic target EsxA (ESAT-6)	Cell wall and cell processes	MDR and XDR
Rv3880c	espL	Function unknown	ESX-1 secretion-associated protein EspL	Cell wall and cell processes	RR, MDR and XDR
Rv3881c	espB	Function unknown. Is a proteolytic substrate of MYCP1|Rv3883c.	Secreted ESX-1 substrate protein B, EspB. Conserved alanine and glycine rich protein	Cell wall and cell processes	RR, MDR and XDR

**Table 3:** Specifically expressed genes in the DR strains compared to the DS strain, sorted in order of gene number.

Locus Tag	Gene Symbol	Function	Product	Functional Category	Strains
Rv0200	Rv0200	Unknown	Possible conserved transmembrane protein	Cell wall and cell processes	RR and MDR
Rv0586	mce2R	Involved in transcriptional mechanism.	Probable transcriptional regulatory protein Mce2R (GntR-family)	Regulatory proteins	RR, MDR, and XDR
Rv0590	mce2B	Unknown, but thought to be involved in host cell invasion.	Mce-family protein Mce2B	Virulence, detoxification, adaptation	RR, MDR, and XDR
Rv0591	mce2C	Unknown, but thought to be involved in host cell invasion	Mce-family protein Mce2C	Virulence, detoxification, adaptation	RR and MDR
Rv1360	Rv1360	Function unknown; probably involved in cellular metabolism	Probable oxidoreductase	Intermediary metabolism and respiration	RR and XDR
Rv1363c	Rv1363c	Function unknown	Possible membrane protein	Cell wall and cell processes	RR and XDR
Rv1368	lprF	Unknown	Probable conserved lipoprotein LprF	Cell wall and cell processes	RR, MDR, and XDR
Rv1595	NadB	Quinolinate biosynthesis	Probable L-aspartate oxidase NadB	Intermediary metabolism and respiration	MDR and XDR
Rv2627c	Rv2627c	Function unknown	Conserved protein	Conserved hypotheticals	RR, MDR, and XDR
Rv2630	Rv2630	Unknown	Hypothetical protein	Conserved hypotheticals	RR and MDR
Rv3323c	moaX	Thought to be involved in molybdenum cofactor biosynthesis.	Probable MoaD-MoaE fusion protein MoaX	Intermediary metabolism and respiration	RR and MDR
Rv3455c	truA	Formation of pseudouridine at positions 38–40 in the anticodon stem and loop of transfer RNAS	Probable tRNA pseudouridine synthase a TruA	Information pathways	RR and MDR

**Table 4:** Undetected genes in all 3 DR strains compared to the DS strain, sorted in order of gene number.

Locus Tag	Gene Symbol	Function	Product	Functional Category
Rv0028	Rv0028	Unknown	Unknown conserved hypothetical protein	Conserved hypotheticals
Rv0103c	ctpB	Cation-transporting ATPase; possibly catalyzes the transport of a cation (possibly copper) with the hydrolyze of ATP	Probable cation-transporter P-type ATPase B CtpB	Cell wall and cell processes
Rv0145	Rv0146	Possible methyltransferase	Possible S-adenosyl methionine-dependent methyltransferase	Lipid metabolism
Rv0203	Rv0203	Unknown	PoctpBssible exported protein	Cell wall and cell processes
Rv0275c	Rv0275c	Could be involved in transcriptional mechanism	Possible transcriptional regulatory protein (possibly TetR-family)	Regulatory proteins
Rv0402c	mmpL1	Unknown. Thought to be involved in fatty acid transport	Probable conserved transmembrane transport protein MmpL1	Cell wall and cell processes
Rv0677c	mmpS5	Unknown	Possible conserved membrane protein MmpS5	Cell wall and cell processes
Rv0855	far	Function unknown, but involvement in lipid degradation (racemization)	Probable fatty-acid-CoA racemase Far	Lipid metabolism
Rv1189	sigI	The sigma factor is an initiation factor that promotes attachment of the RNA polymerase to specific initiation sites and then is released	Possible alternative RNA polymerase sigma factor SigI	Information pathways
Rv1287	Rv1287	Unknown	Conserved hypothetical protein	Conserved hypotheticals
Rv1290c	Rv1290c	Function unknown. Thought to be involved in virulence	Conserved protein	Conserved hypotheticals
Rv1405c	Rv1405c	Causes methylation	Putative methyltransferase	Intermediary metabolism and respiration
Rv1424c	Rv1424c	Unknown	Possible membrane protein	Cell wall and cell processes
Rv1774	Rv1774	Function unknown; probably involved in cellular metabolism	Probable oxidoreductase	Intermediary metabolism and respiration
Rv1841c	Rv1841c	Unknown	Conserved hypothetical membrane protein	Cell wall and cell processes
Rv1976c	Rv1976c	Unknown	Conserved hypothetical protein	Conserved hypotheticals
Rv2116	lppK	Unknown	onserved lipoprotein LppK	Cell wall and cell processes
Rv2219A	Rv2219A	Unknown	Probable conserved membrane protein	Cell wall and cell processes
Rv2370c	Rv2370c	Unknown	Conserved hypothetical protein	Conserved hypotheticals
Rv2383c	mbtB	Involved in the biogenesis of the hydroxyphenyloxazoline-containing siderophore mycobactins. This peptide synthase forms amide bond between the carboxylic acid of salicylate and the alpha-amino group of serine (serine/threonine ligation).	Phenyloxazolinesynthase MbtB (phenyloxazoline synthetase)	Lipid metabolism
Rv2440c	obg	Essential GTP-binding protein	Probable GTP1/Obg-family GTP-binding protein Obg	Intermediary metabolism and respiration
Rv2529	Rv2529	Unknown	Hypothetical protein	Conserved hypotheticals
Rv2633c	Rv2633c	Unknown	Hypothetical protein	Conserved hypotheticals
Rv2714	Rv2714	Unknown	Conserved alanine and leucine rich protein	Conserved hypotheticals
Rv2884	Rv2884	Involved in transcriptional mechanism	Probable transcriptional regulatory protein	Regulatory proteins
Rv2906c	trmD	Specifically methylates guanosime-37 in various TRNAS	Daunorubicin-dim-transport integral membrane protein ABC transporter DrrB	Cell wall and cell processes
Rv2937	drrB	Probably involved in active transport of antibiotic and phthiocerol Dimycocerosate (dim) across the membrane (export). May act jointly to confer daunorubicin and doxorubicin resistance by an export mechanism	Daunorubicin-dim-transport integral membrane protein ABC transporter DrrB	Cell wall and cell processes
Rv2957	Rv2957	Function unknown; probably involved in cellular metabolism	Possible glycosyl transferase	Intermediary metabolism and respiration
Rv2958c	Rv2958c	Unknown; probably involved in cellular metabolism.Possibly involved in resistance to killing by human macrophages	Possible glycosyl transferase	Intermediary metabolism and respiration
Rv3228	Rv3228	Unknown	Conserved hypothetical protein	Conserved hypotheticals
Rv3282	Rv3282	Unknown	Conserved hypothetical protein	Conserved hypotheticals
Rv3321c	vapB44	Unknown	Possible antitoxin VapB44	Virulence, detoxification, adaptation
Rv3330	dacB1	Involved in peptidoglycan synthesis (at final stages). Hydrolyzes the bound d-alanyl-d-alanine	Probablepenicillin-binding protein DacB1 (d-alanyl-d-alanine carboxypeptidase) (DD transpeptidase)	Cell wall and cell processes
Rv3335c	Rv3335c	Unknown	Probable conserved integral membrane protein	Cell wall and cell processes
Rv3338	Rv3338	Unknown	Conserved hypothetical protein	Conserved hypotheticals
Rv3426	PPE58	unknown	PPE family protein PPE58	PE/PPE
Rv3429	PPE59	unknown	PPE family protein PPE59	PE/PPE
Rv3712	Rv3712	Function unknown; probably involved in cellular metabolism	Possible ligase	Intermediary metabolism and respiration
Rv3737	Rv3737	Unknown	Probable conserved transmembrane protein	Cell wall and cell processes
Rv3760	Rv3760	Unknown	Possible conserved membrane protein	Cell wall and cell processes
Rv3824c	papA1	Involved in sulfolipid-1 (SL-1) biosynthesis	Conserved polyketide synthase associated protein PapA1	Lipid metabolism

**Table 5:** Up-regulated genes in the MDR and XDR strains compared to the RR strain, sorted in order of gene number.

Locus Tag	Gene Symbol	Function	Product	Functional Category
Rv2272	Rv2272	Unknown	Probable conserved transmembrane protein	Cell wall and cell processes
Rv2358	smtB	Involved in transcriptional mechanism	Probable transcriptional regulatory protein SmtB (probably ArsR-family)	Regulatory proteins
Rv3244c	lpqB	Unknown	Probable conserved lipoprotein LpqB	Cell wall and cell processes
Rv3339c	Icd1	Involved in the KREBS cycle [catalytic activity: isocitrate + NADP(+) = 2-oxoglutarate + CO(2) + NADPH]	Probable isocitrate dehydrogenase [NADP] Icd1 (oxalosuccinate decarboxylase) (IDH) (NADP^+^-specific ICDH) (IDP)	Intermediary metabolism and respiration
Rv3606c	folK	Involved in dihydrofolate biosynthesis (at the first step) [catalytic activity: ATP + 2-amino-4-hydroxy-6-hydroxymethyl-7,8-dihydropteridine = AMP + 2-amino-7,8-dihydro-4-hydroxy-6-(diphosphooxymethyl)pteridine]	2-amino-4-hydroxy-6-hydroxymethyldihydropteridine pyrophosphokinase FolK (7,8-dihydro-6-hydroxymethylpterin-pyrophosphokinase) (HPPK) (6-hydroxymethyl-7,8-dihydropterin pyrophosphokinase) (PPPK) (2-amino-4-hydroxy-6-hydroxymethyldihydropteridine diphosphokinase) (7,8-dihydro-6-hydroxymethylpterin-diphosphokinase) (6-hydroxymethyl-7,8-dihydropterin diphosphokinase)	Intermediary metabolism and respiration

**Table 6:** Down-regulated genes in the MDR and XDR strains compared to the RR strain, sorted in order of gene number.

Locus Tag	Gene Symbol	Function	Product	Functional Category
Rv1197	esxK	Function unknown	ESAT-6 like protein EsxK (ESAT-6 like protein 3)	Cell wall and cell processes
Rv1361c	PPE19	Function unknown	PPE family protein PPE19	PE/PPE
Rv1534	Rv1534	Possibly involved in a transcriptional mechanism	Probable transcriptional regulator	Regulatory proteins
Rv1642	rpmI	Translation	50S ribosomal protein L35 RpmI	Information pathways
Rv1848	ureA	Involved in the conversion of urea to NH_3_ [catalytic activity: urea + H_2_O = CO_2_ + 2 NH_3_]	Urease gamma subunit UreA (urea amidohydrolase)	Intermediary metabolism and respiration
Rv1932	tpx	Has antioxidant activity. Could remove peroxides or H(2)O(2)	Probable thiol peroxidase Tpx	Virulence, detoxification, and adaptation
Rv1980c	mpt64	Unknown	Immunogenic protein Mpt64 (antigen Mpt64/MPB64)	Cell wall and cell processes
Rv2882c	frr	Responsible for the release of ribosomes from messenger RNA at the termination of protein biosynthesis. May increase the efficiency of translation by recycling ribosomes from one round of translation to another.	Ribosome recycling factor Frr (ribosome releasing factor) (RRF)	Information pathways
Rv3678c	Rv3678c	Function unknown	Conserved protein	Conserved hypotheticals
Rv3874	esxB	Unknown. Exported protein cotranscribed with Rv3875|MT3989|MTV027.10.	10 kDa culture filtrate antigen EsxB (LHP) (CFP10)	Cell wall and cell processes
Rv3875	esxA	Unknown. Elicits high level of inf-gamma from memory effector cells during the first phase of a protective immune response. Exported protein cotranscribed with Rv3874|MT3988|MTV027.09|LHP|CFP10.	6 kDa early secretory antigenic target EsxA (ESAT-6)	Cell wall and cell processes
Rv3880c	espL	Function unknown	ESX-1 secretion-associated protein EspL	Cell wall and cell processes

**Table 7:** Specifically expressed genes in the MDR and XDR strains compared to the RR strain, sorted in order of gene number.

Locus Tag	Gene Symbol	Function	Product	Functional Category
Rv0101	nrp	Involved in lipid metabolism.	Probable peptide synthetase Nrp (peptide synthase)	Lipid metabolism
Rv0280	PPE3	Function unknown	PPE family protein PPE3	Pe/ppe
Rv0924c	mntH	H(+)-stimulated, highly selective, divalent cation uptake system. Responsible for the translocation of the divalent metal across the membrane.	Divalent cation-transport integral membrane protein MntH (BRAMP) (MRAMP)	Cell wall and cell processes
Rv1188	Rv1188	Oxidizes proline to glutamate for use as a carbon and nitrogen source [catalytic activity: l-proline + acceptor + H_2_O = (*S*)-1-pyrroline-5-carboxylate + reduced acceptor]	Probable proline dehydrogenase	Intermediary metabolism and respiration
Rv1473	Rv1473	Thought to be involved in active transport of macrolide across the membrane (export). Macrolide antibiotics resistance by an export mechanism. Responsible for energy coupling to the transport system.	Probable macrolide-transport ATP-binding protein ABC transporter	Cell wall and cell processes
Rv1595	nadB	Quinolinate biosynthesis. Catalyzes the oxidation of l-aspartate to iminoaspartate which is condensed with dihydroxyacetone phosphate to quinolinate under the action of quinolinate synthase a [catalytic activity: L-aspartate + H(2)O + O(2) = oxaloacetate + NH(3) + H(2)O(2)]	Probable l-aspartate oxidase NadB	Intermediary metabolism and respiration
Rv2108	PPE36	Function unknown	PPE family protein PPE36	PE/PPE
Rv3283	sseA	Possibly a sulfotransferase involved in the formation of thiosulfate [catalytic activity: thiosulfate + cyanide = sulfite + thiocyanate].	Probable thiosulfate sulfurtransferase SseA (rhodanese) (thiosulfate cyanide transsulfurase) (thiosulfate thiotransferase)	Intermediary metabolism and respiration

## Data Availability

The data used to support the findings of this study are available from the corresponding author upon request.

## Supplementary Materials

The following are available online at https://www.mdpi.com/article/10.3390/pathogens10050607/s1, Figure S1: Functional distribution of the unexpressed proteins in both MDR and XDR strains, when compared to the RR strain, according to the TubercuList Functional Category (http://svitsrv8.epfl.ch/tuberculist/, accessed on 18 February 2021), Figure S2: Functional distribution of the specially expressed proteins in either the XDR (A) or MDR (B) strains, according to the TubercuList Functional Category (http://svitsrv8.epfl.ch/tuberculist/, accessed on 18 February 2021), Figure S3: The interaction network of the differentially expressed proteins in the DR vs. DS strains and other predicted functional partners, Table S1: The DST patterns of the RR-TB, MDR-TB and XDR-TB clinical isolates, Table S2: Biological Process enrichment in the PPI network of the up-regulated proteins of DR strains and other predicted functional partners, Table S3: Molecular Function enrichment in the PPI network of the up-regulated proteins of DR strains and other predicted functional partners, Table S4: Cellular Component enrichment in the PPI network of the up-regulated proteins of DR strains and other predicted functional partners, Table S5: Biological Process enrichment in the PPI network of the down-regulated proteins of DR strains and other predicted functional partners, Table S6: Molecular Function enrichment in the PPI network of the down-regulated proteins of DR strains and other predicted functional partners, Table S7: Cellular Component enrichment in the PPI network of the down-regulated proteins of DR strains and other predicted functional partners, Table S8: Biological Process enrichment in the PPI network of the specific expressed proteins of DR strains and other predicted functional partners, Table S9: Cellular Component enrichment in the PPI network of the specific expressed proteins of DR strains and other predicted functional partners, Table S10: Biological Process enrichment in the PPI network of the unexpressed proteins of DR strains and other predicted functional partners, Table S11: Cellular Component enrichment in the PPI network of the unexpressed proteins of DR strains and other predicted functional partners.

## Abbreviations

TB, tuberculosis; BCG, Bacillus Calmette-Guerin; MDR-TB, multidrug-resistant TB; XDR-TB, extensively drug-resistant TB; INH, isoniazid; RIF, rifampicin; RR, rifampicin-resistant; DS, drug-sensitive; LFQ, label-free quantification; FASP, filter-aided sample preparation; DST, drug susceptibility testing.
