# Supplementary material for: Label-Free Comparative Proteomics of Differentially Expressed Mycobacterium tuberculosis Protein in Rifampicin-Related Drug-Resistant Strains
Source: Pathogens. 2021 May 15;10(5):607. doi: 10.3390/pathogens10050607 (PMC8157059; doi:10.3390/pathogens10050607)
Supplement: Supplementary file 1 [file pathogens-10-00607-s001.zip › pathogens-1169818-supplementary.pdf]

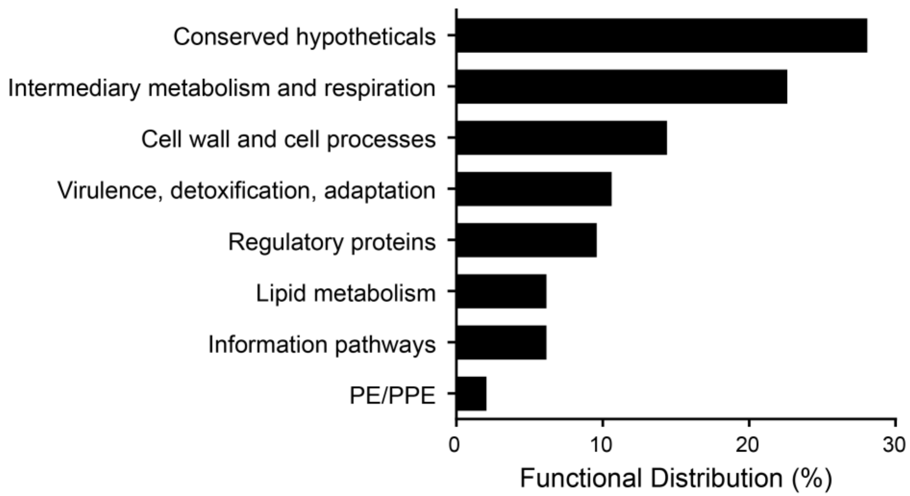

Figure S1. Functional distribution of the unexpressed proteins in both MDR and XDR strains, when compared to the RR strain, according to the TubercuList Functional Category (http://svitsrv8.epfl.ch/tuberculist/). RR, rifampin-resistant; MDR, Multidrug-resistant; XDR, extensively drug-resistant.

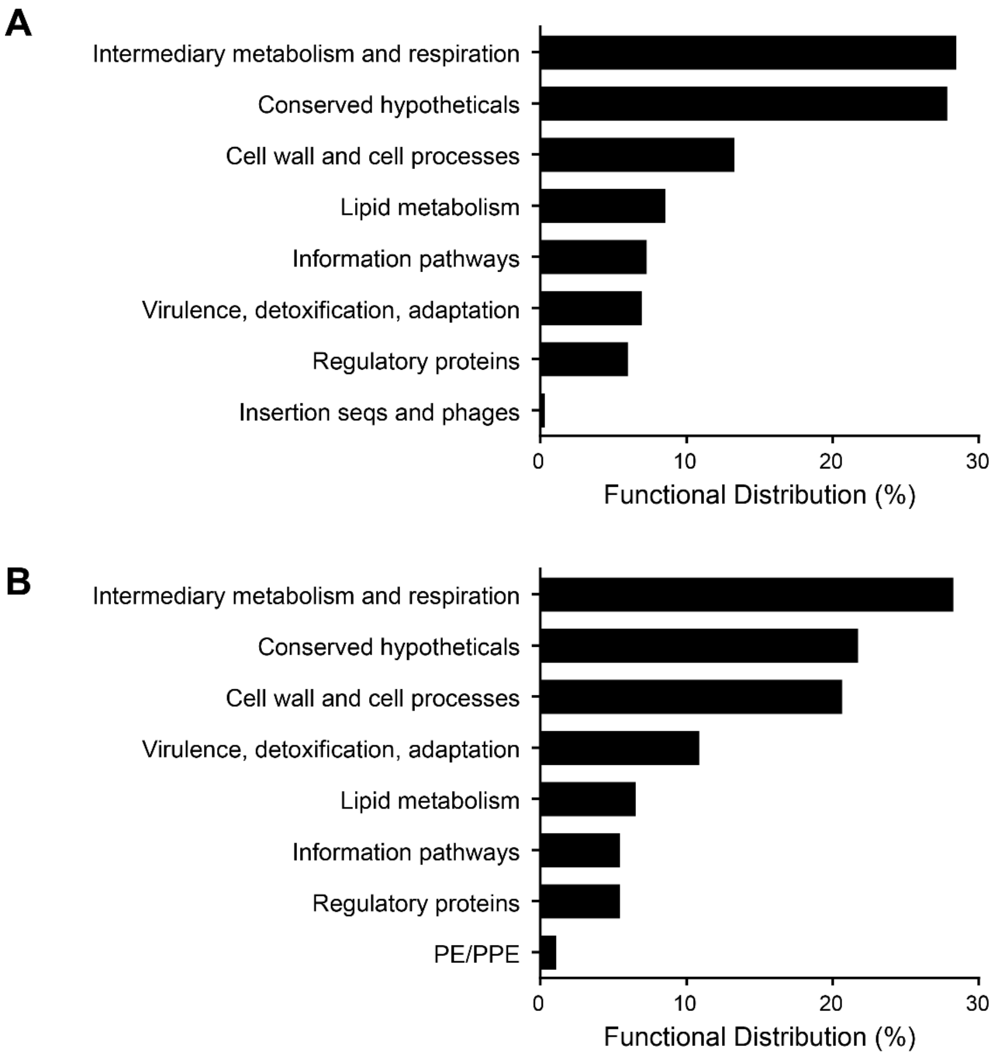

Figure S2. Functional distribution of the specially expressed proteins in either the XDR (**A**) or MDR (**B**) strains, according to the TubercuList Functional Category (http://svitsrv8.epfl.ch/tuberculist/). MDR, Multidrug-resistant; XDR, extensively drug-resistant.

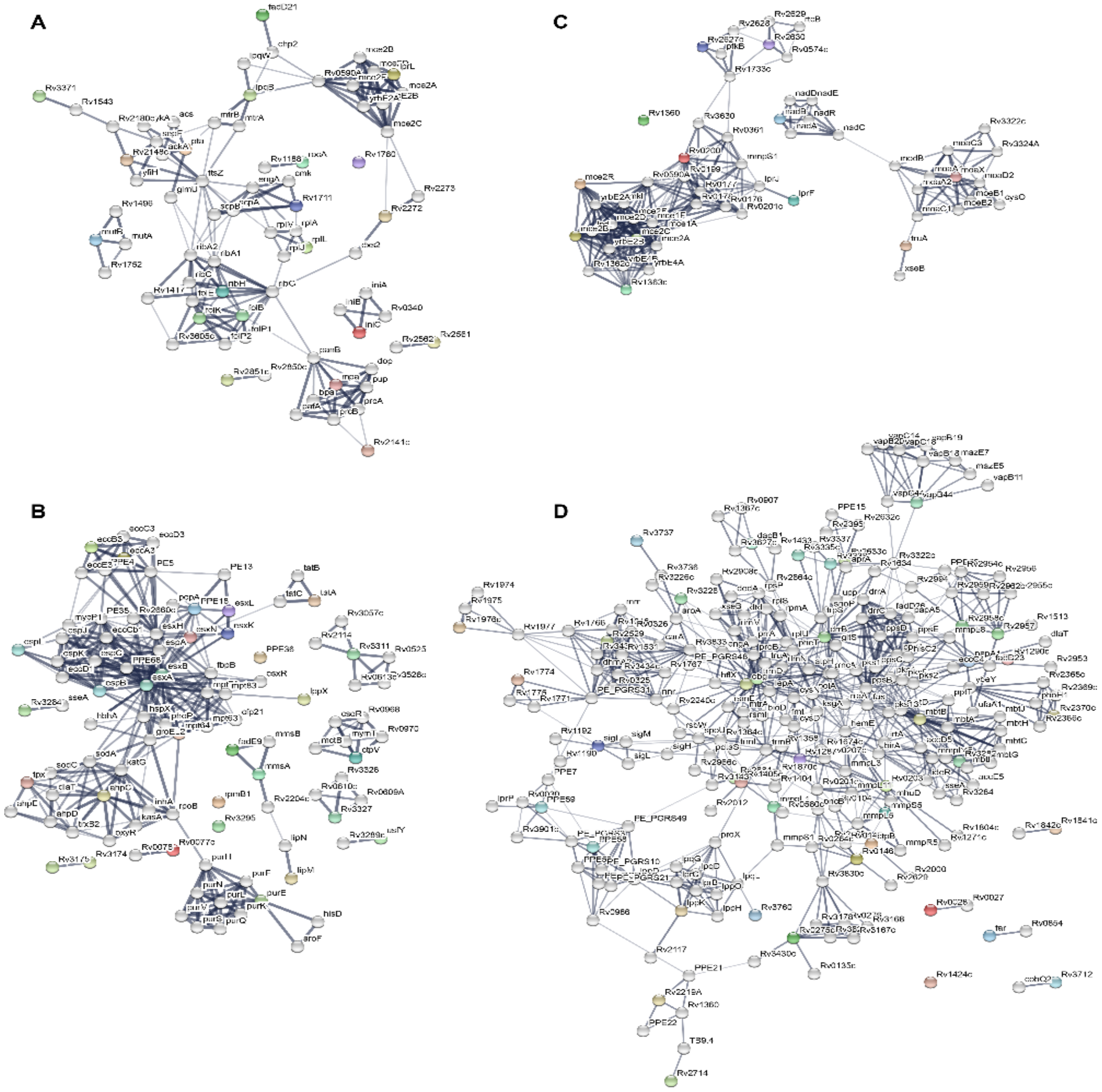

Figure S3. The interaction network of the differentially expressed proteins in the DR vs. DS strains and other predicted functional partners. (**A**) the up-regulated proteins, (**B**) the down-regulated proteins, (**C**) the specifically expressed proteins, and (**D**) the undetectedable proteins in the DR vs. DS strains. The network was gained from the STRING database. Line thickness indicates the strength of data support.

Table S1. The DST patterns of the RR-TB, MDR-TB and XDR-TB clinical isolates.

Drugs	Concentrations (mg/L)	RR-TB	MDR-TB	XDR-TB
RIF	40.0	R	R	R
INH	0.2	S	R	R
EMB	2.0	S	S	R
LFX	2.0	S	S	R
MOX	2.0	S	S	R
KAN	30.0	S	S	R

Table S2. Biological Process enrichment in the PPI network of the up-regulated proteins of DR strains and other predicted functional partners.

#term ID	Description	Observed Gene Count	Background Gene Count	FDR	Matching Proteins
GO:0042364	water-soluble vitamin biosynthetic process	11	44	9.43E-07	Rv1417,cmk,folB,folK,folP1,panB,ribA1,ribA2,ribC,ribG,ribH
GO:1901564	organonitrogen compound metabolic process	31	563	9.43E-07	Rv1417,Rv2851c,ackA,acs,cmk,dop,folB,folE,folK,folP1,folP2,glmU,mpa,mtrA,mtrB,pafA,panB,prcA,prcB,pta,pup,pykA,ribA1,ribA2,ribC,ribG,ribH,rplA,rplJ,rplL,rplM
GO:0010498	proteasomal protein catabolic process	6	6	1.79E-06	dop,mpa,pafA,prcA,prcB,pup
GO:0019941	modification-dependent protein catabolic process	6	6	1.79E-06	dop,mpa,pafA,prcA,prcB,pup
GO:0006771	riboflavin metabolic process	6	7	2.19E-06	Rv1417,ribA1,ribA2,ribC,ribG,ribH
GO:0009231	riboflavin biosynthetic process	6	7	2.19E-06	Rv1417,ribA1,ribA2,ribC,ribG,ribH
GO:0043604	amide biosynthetic process	14	130	5.66E-06	ackA,acs,cmk,folB,folE,folK,folP1,folP2,panB,pta,rplA,rplJ,rplL,rplM
GO:0042398	cellular modified amino acid biosynthetic process	7	21	1.10E-05	cmk,folB,folE,folK,folP1,folP2,panB
GO:0017144	drug metabolic process	14	144	1.50E-05	Rv1417,acs,folB,folE,folK,folP1,mutA,mutB,pykA,ribA1,ribA2,ribC,ribG,ribH
GO:0009108	coenzyme biosynthetic process	11	88	2.62E-05	ackA,acs,cmk,folB,folE,folK,folP1,folP2,panB,pta,pykA
GO:0009396	folic acid-containing compound biosynthetic process	5	7	2.62E-05	folB,folE,folK,folP1,folP2
GO:0006575	cellular modified amino acid metabolic process	7	27	3.52E-05	cmk,folB,folE,folK,folP1,folP2,panB
GO:1901566	organonitrogen compound biosynthetic process	22	401	5.71E-05	Rv1417,ackA,acs,cmk,folB,folE,folK,folP1,folP2,glmU,panB,pta,pykA,ribA1,ribA2,ribC,ribG,ribH,rplA,rplJ,rplL,rplM
GO:0044271	cellular nitrogen compound biosynthetic process	23	443	7.53E-05	Rv1417,ackA,acs,cmk,folB,folE,folK,folP1,folP2,glmU,mtrA,panB,pta,pykA,ribA1,ribA2,ribC,ribG,ribH,rplA,rplJ,rplL,rplM
GO:0006760	folic acid-containing compound metabolic process	5	11	0.00011	folB,folE,folK,folP1,folP2
GO:0046654	tetrahydrofolate biosynthetic process	4	6	0.00029	folB,folE,folK,folP1
GO:0040007	growth	24	541	0.00046	Rv1711,Rv3371,cmk,engA,folB,folE,folP1,ftsZ,glmU,lpqB,lpqW,mtrA,mtrB,panB,prcA,prcB,pup,pykA,ribA2,ribG,ribH,rplJ,rplL,rplM

GO:0044267	cellular protein metabolic process	13	181	0.00047	Rv2851c,dop,mpa,mtrA,mtrB,pafA,prcA,prcB,pup,rplA,rplJ,rplL,rplM
GO:0006807	nitrogen compound metabolic process	32	900	0.001	Rv1417,Rv1711,Rv2851c,ackA,acs,cmk,dop,folB,folE,folK,folP1,folP2,glmU,mpa,mtrA,mtrB,pafA,panB,prcA,prcB,pta,pup,pykA,ribA1,ribA2,ribC,ribG,ribH,rplA,rplJ,rplL,rplM
GO:0018130	heterocycle biosynthetic process	18	355	0.001	Rv1417,ackA,acs,cmk,folB,folE,folK,folP1,folP2,glmU,mtrA,pta,pykA,ribA1,ribA2,ribC,ribG,ribH
GO:0046653	tetrahydrofolate metabolic process	4	10	0.001	folB,folE,folK,folP1
GO:0009987	cellular process	40	1278	0.0011	Rv1417,Rv1711,Rv2851c,Rv3371,ackA,acs,bpa,cmk,dop,fadD21,folB,folE,folK,folP1,folP2,ftsZ,glmU,lpqW,mpa,mtrA,mtrB,mutA,mutB,pafA,panB,prcA,prcB,pta,pup,pykA,ribA1,ribA2,ribC,ribG,ribH,rplA,rplJ,rplL,rplM,sepF
GO:0006084	acetyl-CoA metabolic process	3	3	0.0012	ackA,acs,pta
GO:0006085	acetyl-CoA biosynthetic process	3	3	0.0012	ackA,acs,pta
GO:0071704	organic substance metabolic process	38	1203	0.0015	Rv1417,Rv1711,Rv2851c,Rv3371,ackA,acs,chp2,cmk,dop,fadD21,folB,folE,folK,folP1,folP2,glmU,lpqW,mpa,mtrA,mtrB,mutA,mutB,pafA,panB,prcA,prcB,pta,pup,pykA,ribA1,ribA2,ribC,ribG,ribH,rplA,rplJ,rplL,rplM
GO:0046655	folic acid metabolic process	3	4	0.0019	folB,folK,folP1
GO:0046656	folic acid biosynthetic process	3	4	0.0019	folB,folK,folP1
GO:0070490	protein pupylation	3	4	0.0019	dop,pafA,pup
GO:0044237	cellular metabolic process	37	1182	0.0021	Rv1417,Rv1711,Rv2851c,Rv3371,ackA,acs,cmk,dop,fadD21,folB,folE,folK,folP1,folP2,glmU,lpqW,mpa,mtrA,mtrB,mutA,mutB,pafA,panB,prcA,prcB,pta,pup,pykA,ribA1,ribA2,ribC,ribG,ribH,rplA,rplJ,rplL,rplM
GO:0044283	small molecule biosynthetic process	15	284	0.0021	Rv1417,cmk,fadD21,folB,folE,folK,folP1,folP2,panB,pykA,ribA1,ribA2,ribC,ribG,ribH
GO:1901362	organic cyclic compound biosynthetic process	18	388	0.0021	Rv1417,ackA,acs,cmk,folB,folE,folK,folP1,folP2,glmU,mtrA,pta,pykA,ribA1,ribA2,ribC,ribG,ribH
GO:0044281	small molecule metabolic process	22	552	0.003	Rv1417,Rv3371,ackA,acs,cmk,fadD21,folB,folE,folK,folP1,folP2,glmU,mutA,mutB,panB,pta,pykA,ribA1,ribA2,ribC,ribG,ribH
GO:0043412	macromolecule modification	8	101	0.0052	Rv1711,Rv2851c,dop,mtrA,mtrB,pafA,pup,ribG
GO:0044249	cellular biosynthetic process	26	755	0.0063	Rv1417,Rv3371,ackA,acs,cmk,fadD21,folB,folE,folK,folP1,folP2,glmU,lpqW,mtrA,panB,pta,pykA,ribA1,ribA2,ribC,ribG,ribH,rplA,rplJ,rplL,rplM
GO:0008152	metabolic process	40	1416	0.0072	Rv1417,Rv1543,Rv1711,Rv2851c,Rv3371,ackA,acs,chp2,cmk,dop,fadD21,folB,folE,folK,folP1,folP2,glmU,lpqW,mpa,mtrA,mtrB,mutA,mutB,pafA,panB,prcA,prcB,pta,pup,pykA,ribA1,ribA2,ribC,ribG,ribH,rplA,rplJ,rplL,rplM,yfiH
GO:1901576	organic substance biosynthetic process	26	767	0.0077	Rv1417,Rv3371,ackA,acs,cmk,fadD21,folB,folE,folK,folP1,folP2,glmU,lpqW,mtrA,panB,pta,pykA,ribA1,ribA2,ribC,ribG,ribH,rplA,rplJ,rplL,rplM
GO:0034641	cellular nitrogen compound metabolic process	24	686	0.0083	Rv1417,Rv1711,ackA,acs,cmk,folB,folE,folK,folP1,folP2,glmU,mtrA,panB,pta,pykA,ribA1,ribA2,ribC,ribG,ribH,rplA,rplJ,rplL,rplM
GO:0019678	propionate metabolic process, methylmalonyl pathway	2	2	0.0116	mutA,mutB
GO:0006464	cellular protein modification process	6	71	0.0154	Rv2851c,dop,mtrA,mtrB,pafA,pup

GO:0018193	peptidyl-amino acid modification	4	31	0.0203	dop,mtrB,pafA,pup
GO:0042254	ribosome biogenesis	4	33	0.0245	Rv1711,engA,rplA,rplJ
GO:0044085	cellular component biogenesis	8	139	0.0261	Rv1711,bpa,engA,ftsZ,glmU,rplA,rplJ,sepF
GO:0071732	cellular response to nitric oxide	2	4	0.0261	mpa,pafA
GO:0015940	pantothenate biosynthetic process	2	5	0.0327	cmk,panB
GO:0071731	response to nitric oxide	3	19	0.0348	Rv3371,mpa,pafA
GO:0046483	heterocycle metabolic process	20	608	0.0356	Rv1417,Rv1711,ackA,acs,cmk,folB,folE,folK,folP1,folP2,glmU,mtrA,pta,pykA,ribA1,ribA2,ribC,ribG,ribH,rplA
GO:0043650	dicarboxylic acid biosynthetic process	3	20	0.0382	folB,folK,folP1
GO:0015939	pantothenate metabolic process	2	6	0.0406	cmk,panB
GO:0090407	organophosphate biosynthetic process	8	159	0.048	ackA,acs,cmk,folE,glmU,lpqW,pta,pykA
GO:0016310	phosphorylation	6	97	0.0485	ackA,cmk,folK,mtrA,mtrB,pykA
GO:0006082	organic acid metabolic process	13	343	0.05	ackA,acs,cmk,fadD21,folB,folE,folK,folP1,folP2,mutA,mutB,panB,pykA

Table S3. Molecular Function enrichment in the PPI network of the up-regulated proteins of DR strains and other predicted functional partners.

#term ID	Description	Observed Gene Count	Background Gene Count	FDR	Matching Proteins
GO:0070628	proteasome binding	3	3	0.0141	bpa,mpa,pup
GO:0003933	GTP cyclohydrolase activity	2	2	0.0236	folE,ribA2
GO:0004298	threonine-type endopeptidase activity	2	2	0.0236	prcA,prcB
GO:0004494	methylmalonyl-CoA mutase activity	2	3	0.0236	mutA,mutB
GO:0005525	GTP binding	5	30	0.0236	Rv1496,engA,folE,ftsZ,ribA2
GO:0008144	drug binding	14	331	0.0236	Rv1496,Rv2148c,Rv2850c,ackA,acs,cmk,dop,folK,mpa,mtrB,mutA,mutB,pafA,pykA
GO:0008686	3,4-dihydroxy-2-butanone-4-phosphate synthase activity	2	2	0.0236	ribA1,ribA2
GO:0016814	hydrolase activity, acting on carbon-nitrogen (but not peptide) bonds, in cyclic amidines	3	9	0.0236	folE,ribA2,ribG
GO:0032555	purine ribonucleotide binding	15	312	0.0236	Rv1496,Rv2850c,ackA,acs,cmk,dop,engA,folE,folK,ftsZ,mpa,mtrB,pafA,pykA,ribA2
GO:0035639	purine ribonucleoside triphosphate binding	15	307	0.0236	Rv1496,Rv2850c,ackA,acs,cmk,dop,engA,folE,folK,ftsZ,mpa,mtrB,pafA,pykA,ribA2
GO:0036094	small molecule binding	18	497	0.0245	Rv1496,Rv2148c,Rv2850c,ackA,acs,cmk,dop,engA,folE,folK,ftsZ,mpa,mtrB,mutA,mutB,pafA,pykA,ribA2
GO:0005488	binding	32	1122	0.0248	Rv1496,Rv1711,Rv2148c,Rv2850c,ackA,acs,bpa,cmk,dop,engA,folE,folK,folP1,ftsZ,glmU,mpa,mtrA,mtrB,mutA,mutB,pafA,panB,prcB,pup,pykA,ribA1,ribA2,ribG,rplA,rplJ,rplM,yfiH
GO:0016740	transferase activity	16	440	0.034	Rv2851c,Rv3371,ackA,chp2,cmk,fadD21,folK,folP1,glmU,mtrB,pafA,panB,pta,pykA,ribC,ribH
GO:0031419	cobalamin binding	2	5	0.034	mutA,mutB
GO:0043167	ion binding	25	821	0.034	Rv1496,Rv2148c,Rv2850c,ackA,acs,cmk,dop,engA,folE,folK,folP1,ftsZ,glmU,mpa,mtrA,mtrB,mut

					A,mutB,pafA,panB,pykA,ribA1,ribA2,ribG,yfiH
GO:0016810	hydrolase activity, acting on carbon-nitrogen (but not peptide) bonds	4	41	0.0382	dop,folE,ribA2,ribG
GO:0046872	metal ion binding	17	490	0.0382	ackA,acs,dop,folE,folP1,ftsZ,glmU,mtrA,mutA,mutB,pafA,panB,pykA,ribA1,ribA2,ribG,yfiH
GO:0016301	kinase activity	5	67	0.0389	ackA,cmk,folK,mtrB,pykA
GO:0016772	transferase activity, transferring phosphorus-containing groups	7	127	0.0389	ackA,cmk,fadD21,folK,glmU,mtrB,pykA
GO:0016866	intramolecular transferase activity	3	23	0.0389	Rv1711,mutA,mutB
GO:0043168	anion binding	16	465	0.0389	Rv1496,Rv2148c,Rv2850c,ackA,acs,cmk,dop,engA,folE,folK,ftsZ,mpa,mtrB,pafA,pykA,ribA2
GO:0097159	organic cyclic compound binding	23	756	0.0389	Rv1496,Rv1711,Rv2148c,Rv2850c,ackA,acs,cmk,dop,engA,folE,folK,ftsZ,mpa,mtrA,mtrB,mutA,mutB,pafA,pykA,ribA2,rplA,rplJ,rplM
GO:1901363	heterocyclic compound binding	23	756	0.0389	Rv1496,Rv1711,Rv2148c,Rv2850c,ackA,acs,cmk,dop,engA,folE,folK,ftsZ,mpa,mtrA,mtrB,mutA,mutB,pafA,pykA,ribA2,rplA,rplJ,rplM
GO:0003824	catalytic activity	34	1310	0.0477	Rv1496,Rv1543,Rv1711,Rv2850c,Rv2851c,Rv3371,ackA,acs,chp2,cmk,dop,fadD21,folB,folE,folK,folP1,ftsZ,glmU,mpa,mtrB,mutA,mutB,pafA,panB,prcA,prcB,pta,pykA,ribA1,ribA2,ribC,ribG,ribH,yfiH

Table S4. Cellular Component enrichment in the PPI network of the up-regulated proteins of DR strains and other predicted functional partners.

#term ID	Description	Observed Gene Count	Background Gene Count	FDR	Matching Proteins
GO:0000502	proteasome complex	4	4	0.00028	bpa,mpa,prcA,prcB
GO:0005622	intracellular	28	781	0.0027	Rv1711,Rv2148c,ackA,bpa,cmk,folE,folP1,folP2,ftsZ,glmU,iniC,mpa,mtrA,mtrB,mutA,panB,prcA,prcB,pta,pykA,ribC,ribG,ribH,rplA,rplJ,rplL,rplM,sepF
GO:0044424	intracellular part	27	757	0.0033	Rv1711,ackA,bpa,cmk,folE,folP1,folP2,ftsZ,glmU,iniC,mpa,mtrA,mtrB,mutA,panB,prcA,prcB,pta,pykA,ribC,ribG,ribH,rplA,rplJ,rplL,rplM,sepF
GO:0044464	cell part	43	1541	0.0049	Rv1417,Rv1543,Rv1711,Rv2148c,Rv2272,Rv2273,Rv3371,ackA,acs,bpa,chp2,cmk,engA,fadD21,folE,folP1,folP2,ftsZ,glmU,iniA,iniC,lpqB,lpqW,mpa,mtrA,mtrB,mutA,mutB,pafA,panB,prcA,prcB,pta,pykA,ribC,ribG,ribH,rplA,rplJ,rplL,rplM,sepF,yfiH
GO:0005737	cytoplasm	25	729	0.0066	Rv1711,ackA,cmk,folE,folP1,folP2,ftsZ,glmU,iniC,mtrA,mtrB,mutA,panB,prcA,prcB,pta,pykA,ribC,ribG,ribH,rplA,rplJ,rplL,rplM,sepF
GO:0005839	proteasome core complex	2	2	0.0088	prcA,prcB
GO:0022624	proteasome accessory complex	2	2	0.0088	bpa,mpa
GO:0032991	protein-containing complex	9	184	0.0297	bpa,mpa,prcA,prcB,ribH,rplA,rplJ,rplL,rplM
GO:0005829	cytosol	15	416	0.0339	Rv1711,cmk,folP1,folP2,iniC,mtrB,mutA,pykA,ribC,ribG,ribH,rplA,rplJ,rplL,rplM
GO:0005886	plasma membrane	30	1091	0.0341	Rv1417,Rv1543,Rv2272,Rv2273,Rv3371,acs,bpa,chp2,engA,fadD21,ftsZ,iniA,iniC,lpqB,mpa,mtrA,mtrB,mutA,mutB,pafA,panB,prcA,prcB,pykA,ribG,rplA,rplJ,rplL,rplM,sepF

GO:1902494	catalytic complex	5	69	0.0341	bpa,mpa,prcA,prcB,ribH
GO:0022625	cytosolic large ribosomal subunit	3	26	0.0417	rplA,rplJ,rplM
GO:0005618	cell wall	17	529	0.0423	Rv1543,Rv3371,acs,bpa,chp2,engA,iniA,lpqB,mpa,mutA,pafA,prcA,rplA,rplJ,rplL,rplM,yfiH

Table S5. Biological Process enrichment in the PPI network of the down-regulated proteins of DR strains and other predicted functional partners.

#term ID	Description	Observed Gene Count	Background Gene Count	FDR	Matching Proteins
GO:0044419	interspecies interaction between organisms	31	301	6.22E-09	PE13,PE35,PE5,PPE18,PPE68,ahpC,ahpE,ctpV,dlaT,eccCb1,eccD1,espA,espB,espC,espJ,espL,esxA,esxB,esxH,esxL,fbpB,groEL2,hbhA,hspX,katG,lppX,mpt83,mycP1,sodA,sodC,tpx
GO:0009405	pathogenesis	22	160	2.66E-08	PE13,PE35,PE5,PPE18,PPE68,ctpV,dlaT,eccCb1,espA,espB,espC,espJ,esxA,esxB,esxH,esxL,hbhA,katG,lppX,mycP1,sodA,tpx
GO:0098869	cellular oxidant detoxification	9	17	5.62E-07	ahpC,ahpD,ahpE,dlaT,katG,sodA,sodC,tpx,trxB2
GO:0006189	'de novo' IMP biosynthetic process	8	12	7.48E-07	purE,purF,purH,purK,purL,purM,purN,purQ
GO:0009306	protein secretion	8	14	1.09E-06	eccCb1,espA,espB,espC,esxA,esxB,tatA,tatB
GO:0046040	IMP metabolic process	8	15	1.37E-06	purE,purF,purH,purK,purL,purM,purN,purQ
GO:0020012	evasion or tolerance of host immune response	9	25	2.47E-06	ahpC,ahpE,eccCb1,eccD1,espC,katG,sodA,sodC,tpx
GO:0044413	avoidance of host defenses	10	35	2.47E-06	ahpC,ahpE,eccCb1,eccD1,espC,espL,katG,sodA,sodC,tpx
GO:0071806	protein transmembrane transport	8	17	2.47E-06	eccCb1,espA,espB,espC,esxA,esxB,tatB,tatC
GO:0015031	protein transport	9	29	3.69E-06	eccCb1,espA,espB,espC,esxA,esxB,tatA,tatB,tatC
GO:0033036	macromolecule localization	10	41	4.19E-06	eccCb1,espA,espB,espC,esxA,esxB,lppX,tatA,tatB,tatC
GO:0052572	response to host immune response	14	96	4.23E-06	ahpC,ahpE,eccCb1,eccD1,espC,espL,esxA,fbpB,hspX,katG,mpt83,sodA,sodC,tpx
GO:0042592	homeostatic process	10	43	5.26E-06	ahpC,ahpD,ahpE,dlaT,eccA3,mctB,mymT,sodC,tpx,trxB2
GO:0070887	cellular response to chemical stimulus	10	43	5.26E-06	ahpC,ahpD,ahpE,dlaT,hspX,katG,sodA,sodC,tpx,trxB2
GO:0051701	interaction with host	15	119	7.06E-06	ahpC,ahpE,eccCb1,eccD1,espC,espL,esxA,fbpB,hbhA,hspX,katG,mpt83,sodA,sodC,tpx
GO:0044315	protein secretion by the type VII secretion system	6	9	9.46E-06	eccCb1,espA,espB,espC,esxA,esxB
GO:0009605	response to external stimulus	16	144	1.24E-05	ahpC,ahpE,eccA3,eccCb1,eccD1,espC,espL,esxA,fbpB,hspX,katG,mpt64,mpt83,sodA,sodC,tpx
GO:0042221	response to chemical	16	153	2.37E-05	ahpC,ahpD,ahpE,csoR,ctpV,dlaT,fbpB,hspX,inhA,katG,mymT,rpoB,sodA,sodC,tpx,trxB2
GO:0045454	cell redox homeostasis	7	21	3.33E-05	ahpC,ahpD,ahpE,dlaT,sodC,tpx,trxB2
GO:0071702	organic substance transport	10	56	3.33E-05	eccCb1,espA,espB,espC,esxA,esxB,lppX,tatA,tatB,tatC
GO:0009168	purine ribonucleoside monophosphate biosynthetic process	9	45	4.54E-05	dlaT,purE,purF,purH,purK,purL,purM,purN,purQ
GO:0044403	symbiont process	17	187	5.68E-05	ahpC,ahpE,eccCb1,eccD1,espC,espL,esxA,fbpB,groEL2,hbhA,hspX,katG,lppX,mpt83,sodA,sodC,tpx
GO:0019725	cellular homeostasis	8	39	0.00013	ahpC,ahpD,ahpE,dlaT,eccA3,sodC,tpx,trxB2
GO:0009167	purine ribonucleoside monophosphate metabolic process	9	54	0.00016	dlaT,purE,purF,purH,purK,purL,purM,purN,purQ

GO:0050896	response to stimulus	25	401	0.00016	ahpC,ahpD,ahpE,csoR,ctpV,dlaT,eccA3,eccCb1,eccD1,espC,espL,esxA,fbpB,groEL2,hspX,inhA,katG,mpt64,mpt83,mymT,rpoB,sodA,sodC,tpx,trxB2
GO:0052059	evasion or tolerance by symbiont of host-produced reactive oxygen species	4	4	0.00019	ahpC,katG,sodA,sodC
GO:0072593	reactive oxygen species metabolic process	4	5	0.00032	katG,sodA,sodC,trxB2
GO:0009152	purine ribonucleotide biosynthetic process	9	63	0.0004	dlaT,purE,purF,purH,purK,purL,purM,purN,purQ
GO:0065008	regulation of biological quality	11	97	0.0004	Rv3057c,ahpC,ahpD,ahpE,dlaT,eccA3,mctB,mymT,sodC,tpx,trxB2
GO:0010035	response to inorganic substance	8	49	0.00045	csoR,ctpV,hspX,katG,mymT,sodA,sodC,trxB2
GO:0052060	evasion or tolerance by symbiont of host-produced nitric oxide	4	6	0.00048	ahpC,ahpE,sodC,tpx
GO:0006810	transport	13	158	0.0014	ctpV,eccA3,eccCb1,espA,espB,espC,esxA,esxB,lppX,mctB,tatA,tatB,tatC
GO:0019430	removal of superoxide radicals	3	3	0.0017	sodA,sodC,trxB2
GO:0040007	growth	27	541	0.0019	PPE4,Rv0525,aroF,dlaT,eccA3,eccB3,eccC3,eccCb1,eccD1,eccD3,eccE3,fbpB,groEL2,hisD,hspX,kasA,lppX,purE,purF,purH,purL,purQ,rpoB,tatA,tatB,tatC,trxB2
GO:0034614	cellular response to reactive oxygen species	4	11	0.0022	katG,sodA,sodC,trxB2
GO:0009150	purine ribonucleotide metabolic process	9	85	0.0024	dlaT,purE,purF,purH,purK,purL,purM,purN,purQ
GO:0051716	cellular response to stimulus	12	167	0.0063	ahpC,ahpD,ahpE,dlaT,eccA3,hspX,katG,mpt64,sodA,sodC,tpx,trxB2
GO:0051409	response to nitrosative stress	4	16	0.0064	ahpC,ahpE,hspX,tpx
GO:0055085	transmembrane transport	10	123	0.0068	ctpV,eccCb1,espA,espB,espC,esxA,esxB,mctB,tatB,tatC
GO:0046688	response to copper ion	3	7	0.0076	csoR,ctpV,mymT
GO:0010038	response to metal ion	4	19	0.0103	csoR,ctpV,hspX,mymT
GO:0006979	response to oxidative stress	6	50	0.0107	ahpD,katG,sodA,sodC,tpx,trxB2
GO:0043953	protein transport by the Tat complex	2	2	0.0169	tatB,tatC
GO:0055070	copper ion homeostasis	2	2	0.0169	mctB,mymT
GO:0006950	response to stress	12	197	0.0206	ahpC,ahpD,ahpE,eccA3,groEL2,hspX,katG,mpt64,sodA,sodC,tpx,trxB2
GO:0065007	biological regulation	19	398	0.0224	Rv3057c,ahpC,ahpD,ahpE,csoR,dlaT,eccA3,espL,fbpB,groEL2,hbhA,hspX,katG,mctB,mycP1,mymT,sodC,tpx,trxB2
GO:0042783	active evasion of host immune response	3	14	0.0349	eccCb1,eccD1,espC
GO:0055076	transition metal ion homeostasis	3	16	0.0463	eccA3,mctB,mymT

Table S6. Molecular Function enrichment in the PPI network of the down-regulated proteins of DR strains and other predicted functional partners.

#term ID	Description	Observed Gene Count	Background Gene Count	FDR	Matching Proteins
GO:0016209	antioxidant activity	9	17	8.88E-07	ahpC,ahpD,ahpE,dlaT,katG,sodA,sodC,tpx,trxB2
GO:0004601	peroxidase activity	5	9	0.0013	ahpC,ahpD,ahpE,katG,tpx
GO:0051920	peroxiredoxin activity	4	6	0.003	ahpC,ahpD,ahpE,tpx

GO:0016667	oxidoreductase activity, acting on a sulfur group of donors	5	19	0.0075	ahpC,ahpD,dlaT,tpx,trxB2
GO:0051287	NAD binding	6	30	0.0075	aroF,hisD,inhA,katG,mmsA,mmsB
GO:0015036	disulfide oxidoreductase activity	4	12	0.0134	ahpD,dlaT,tpx,trxB2
GO:0016668	oxidoreductase activity, acting on a sulfur group of donors, NAD(P) as acceptor	3	8	0.0492	ahpC,dlaT,trxB2

Table S7. Cellular Component enrichment in the PPI network of the down-regulated proteins of DR strains and other predicted functional partners.

#term ID	Description	Observed Gene Count	Background Gene Count	FDR	Matching Proteins
GO:0005576	extracellular region	30	287	1.77E-09	PE13,PE35,PE5,PPE18,PPE68,cfp21,eccB3,espA,espB,espC,espJ,esxA,esxB,esxH,esxK,esxL,esxN,esxR,fbpB,groEL2,hspX,katG,lppX,mpt63,mpt64,mpt70,mpt83,sodA,sodC,tpx
GO:0030312	external encapsulating structure	37	532	1.44E-07	PE13,PPE18,PPE68,Rv0968,ahpC,cfp21,ctpV,dlaT,eccA3,eccB3,eccC3,eccCb1,eccD1,eccD3,eccE3,espC,espL,esxA,esxB,esxL,fbpB,groEL2,hbhA,hisD,hspX,inhA,kasA,katG,lppX,mctB,mpt63,mpt64,mpt83,rpoB,sodC,sseA,tpx
GO:0005618	cell wall	36	529	3.04E-07	PE13,PPE18,PPE68,Rv0968,ahpC,cfp21,ctpV,dlaT,eccA3,eccB3,eccC3,eccCb1,eccD1,eccD3,eccE3,espC,espL,esxA,esxB,esxL,fbpB,groEL2,hbhA,hisD,hspX,inhA,kasA,katG,lppX,mpt63,mpt64,mpt83,rpoB,sodC,sseA,tpx
GO:0044464	cell part	66	1541	6.00E-07	PE13,PE35,PE5,PPE18,PPE36,PPE4,PPE68,Rv0525,Rv0968,Rv0970,Rv2204c,Rv3057c,ahpC,ahpD,ahpE,aroF,cfp21,csoR,ctpV,dlaT,eccA3,eccB3,eccC3,eccCb1,eccD1,eccD3,eccE3,espA,espC,espK,espL,esxA,esxB,esxL,esxN,fbpB,groEL2,hbhA,hisD,hspX,inhA,kasA,katG,lppX,mctB,mmsA,mpt63,mpt64,mpt70,mpt83,mycP1,purH,purK,purL,purM,purQ,rpmB1,rpoB,sodA,sodC,sseA,tatA,tatB,tatC,tpx,trxB2
GO:0071944	cell periphery	55	1224	5.72E-06	PE13,PPE18,PPE36,PPE4,PPE68,Rv0525,Rv0968,Rv0970,Rv2204c,ahpC,ahpD,cfp21,csoR,ctpV,dlaT,eccA3,eccB3,eccC3,eccCb1,eccD1,eccD3,eccE3,espA,espC,espK,espL,esxA,esxB,esxL,esxN,fbpB,groEL2,hbhA,hisD,hspX,inhA,kasA,katG,lppX,mctB,mpt63,mpt64,mpt83,mycP1,purH,purK,purQ,rpoB,sodA,sodC,sseA,tatA,tatB,tatC,tpx
GO:0005615	extracellular space	5	8	0.0001	espA,mpt63,mpt70,mpt83,sodC
GO:0005886	plasma membrane	46	1091	0.00043	PPE36,PPE4,PPE68,Rv0525,Rv0968,Rv0970,Rv2204c,ahpC,ahpD,csoR,ctpV,dlaT,eccA3,eccB3,eccC3,eccCb1,eccD1,eccD3,eccE3,espA,espK,espL,esxA,esxB,esxL,esxN,fbpB,groEL2,hbhA,hspX,inhA,kasA,katG,lppX,mpt83,mycP1,purH,purK,purQ,rpoB,sodA,sodC,sseA,tatA,tatB,tatC
GO:0009986	cell surface	7	34	0.00043	PE35,PE5,PPE18,PPE68,hbhA,lppX,mycP1
GO:0016020	membrane	47	1122	0.00043	PPE36,PPE4,PPE68,Rv0525,Rv0968,Rv0970,Rv2204c,ahpC,ahpD,csoR,ctpV,dlaT,eccA3,eccB3,eccC3,eccCb1,eccD1,eccD3,eccE3,espA,espK,espL,esxA,esxB,esxL,esxN,fbpB,groEL2,hbhA,hspX,inhA,kasA,katG,lppX,mctB,mpt83,mycP1,purH,purK,purQ,rpoB,sodA,sodC,sseA,tatA,tatB,tatC
GO:0005829	cytosol	24	416	0.00062	Rv2204c,Rv3057c,ahpC,ahpD,ahpE,aroF,dlaT,eccA3,espK,groEL2,hbhA,hisD,hspX,kasA,katG,purK,purM,rpmB1,rpoB,sodA,sseA,tatC,tpx,trxB2
GO:0044444	cytoplasmic part	25	483	0.0021	Rv2204c,Rv3057c,ahpC,ahpD,ahpE,aroF,dlaT,eccA3,espK,groEL2,hbhA,hisD,hspX,kasA,katG,mm

					sA,purK,purM,rpmB1,rpoB,sodA,sseA,tatC,tpx,trxB2
GO:0031975	envelope	5	32	0.0108	PE5,groEL2,mctB,mpt70,mpt83
GO:0030313	cell envelope	4	19	0.0114	groEL2,mctB,mpt70,mpt83
GO:0005737	cytoplasm	30	729	0.0158	Rv2204c,Rv3057c,ahpC,ahpD,ahpE,aroF,csoR,dlaT,eccA3,eccCb1,espK,esxA,groEL2,hbhA,hisD,hspX,kasA,katG,mmsA,purK,purL,purM,purQ,rpmB1,rpoB,sodA,sseA,tatC,tpx,trxB2
GO:0044165	host cell endoplasmic reticulum	2	2	0.0169	esxA,esxB
GO:0044228	host cell surface	2	3	0.0248	esxA,esxB
GO:0042597	periplasmic space	3	15	0.035	mpt70,mpt83,sodA

Table S8. Biological Process enrichment in the PPI network of the specific expressed proteins of DR strains and other predicted functional partners.

#term ID	Description	Observed Gene Count	Background Gene Count	FDR	Matching Proteins
GO:0009108	coenzyme biosynthetic process	11	88	1.99E-05	moaA1,moaA2,moaC1,moaC3,moeB2,nadA,nadB,nadC,nadD,nadE,pfkB
GO:0009435	NAD biosynthetic process	5	7	4.57E-05	nadA,nadB,nadC,nadD,nadE
GO:0019674	NAD metabolic process	5	9	5.65E-05	nadA,nadB,nadC,nadD,nadE
GO:0019359	nicotinamide nucleotide biosynthetic process	6	22	9.42E-05	nadA,nadB,nadC,nadD,nadE,pfkB
GO:0006777	Mo-molybdopterin cofactor biosynthetic process	5	13	0.00012	moaA1,moaA2,moaC1,moaC3,moeB2
GO:0019720	Mo-molybdopterin cofactor metabolic process	5	13	0.00012	moaA1,moaA2,moaC1,moaC3,moeB2
GO:0046496	nicotinamide nucleotide metabolic process	6	30	0.00017	nadA,nadB,nadC,nadD,nadE,pfkB
GO:0090407	organophosphate biosynthetic process	11	159	0.00027	moaA1,moaA2,moaC1,moaC3,moeB2,nadA,nadB,nadC,nadD,nadE,pfkB
GO:0019637	organophosphate metabolic process	11	205	0.0022	moaA1,moaA2,moaC1,moaC3,moeB2,nadA,nadB,nadC,nadD,nadE,pfkB
GO:0006531	aspartate metabolic process	2	2	0.0158	nadA,nadD
GO:0006796	phosphate-containing compound metabolic process	11	269	0.0158	moaA1,moaA2,moaC1,moaC3,moeB2,nadA,nadB,nadC,nadD,nadE,pfkB
GO:0034628	'de novo' NAD biosynthetic process from aspartate	2	2	0.0158	nadA,nadD
GO:0046874	quinolinate metabolic process	2	2	0.0158	nadA,nadC
GO:0018130	heterocycle biosynthetic process	12	355	0.0376	mce2R,moaA1,moaA2,moaC1,moaC3,moeB2,nadA,nadB,nadC,nadD,nadE,pfkB
GO:1901566	organonitrogen compound biosynthetic process	13	401	0.0376	cysO,moaA1,moaA2,moaC1,moaC3,moeB1,moeB2,nadA,nadB,nadC,nadD,nadE,pfkB

Table S9. Cellular Component enrichment in the PPI network of the specific expressed proteins of DR strains and other predicted functional partners.

#term ID	Description	Observed Gene Count	Background Gene Count	FDR	Matching Proteins
GO:0019008	molybdopterin synthase complex	2	2	0.0237	moaA1,moaA2

Table S10. Biological Process enrichment in the PPI network of the unexpressed proteins of DR strains and other predicted functional partners.

#term ID	Description	Observed Gene Count	Background Gene Count	FDR	Matching Proteins
GO:0071770	DIM/DIP cell wall layer assembly	11	19	0.00024	Rv2953,Rv2959c,drrB,fadD26,papA5,pks1,ppsA,ppsB,ppsC,ppsD,ppsE
GO:0018958	phenol-containing compound metabolic process	8	14	0.0052	ideR,mbtB,mbtG,mbtI,ppsA,ppsB,ppsC,ppsD
GO:0034660	ncRNA metabolic process	16	68	0.0052	dtd,fmt,gltS,ksgA,pheT,rimM,rlmN,rnc,rsmE,rsmI,trmB,trmD,trmI,trpS,truA,ybeY
GO:0071766	Actinobacterium-type cell wall biogenesis	13	49	0.0057	Rv2953,Rv2959c,drrB,fadD23,fadD26,mmpL3,papA5,pks1,ppsA,ppsB,ppsC,ppsD,ppsE
GO:0034470	ncRNA processing	12	43	0.006	fmt,ksgA,rimM,rlmN,rnc,rsmE,rsmI,trmB,trmD,trmI,truA,ybeY
GO:0001510	RNA methylation	7	14	0.0104	ksgA,rlmN,rsmE,rsmI,trmB,trmD,trmI
GO:0044085	cellular component biogenesis	22	139	0.0104	Rv1433,Rv2953,Rv2959c,drrB,engA,fadD23,fadD26,ksgA,mmpL3,papA5,pks1,ppsA,ppsB,ppsC,ppsD,ppsE,rimM,rlmN,rnc,rsmE,rsmI,ybeY
GO:0009451	RNA modification	9	30	0.019	fmt,ksgA,rlmN,rsmE,rsmI,trmB,trmD,trmI,truA
GO:0006364	rRNA processing	7	21	0.0194	ksgA,rimM,rlmN,rnc,rsmE,rsmI,ybeY
GO:0006399	tRNA metabolic process	11	47	0.0194	dtd,fmt,gltS,pheT,rlmN,rnc,trmB,trmD,trmI,trpS,truA
GO:0006633	fatty acid biosynthetic process	13	64	0.0194	fadD23,fadD26,mbtB,pks1,pks2,pks4,pks5,ppsA,ppsB,ppsC,ppsD,ppsE,ufaA1
GO:0008610	lipid biosynthetic process	22	155	0.0194	Rv2953,Rv2957,Rv2958c,Rv2959c,Rv2962c,drrB,fadD23,fadD26,mbtB,mmpL8,papA1,papA5,pks1,pks2,pks4,pks5,ppsA,ppsB,ppsC,ppsD,ppsE,ufaA1
GO:0009273	peptidoglycan-based cell wall biogenesis	14	74	0.0194	Rv1433,Rv2953,Rv2959c,drrB,fadD23,fadD26,mmpL3,papA5,pks1,ppsA,ppsB,ppsC,ppsD,ppsE
GO:0030488	tRNA methylation	4	4	0.0194	rlmN,trmB,trmD,trmI
GO:0032259	methylation	13	65	0.0194	Rv0145,Rv0146,Rv0560c,Rv1405c,Rv2959c,ksgA,rlmN,rsmE,rsmI,trmB,trmD,trmI,ufaA1
GO:0042844	glycol metabolic process	5	7	0.0194	fadD26,ppsA,ppsB,ppsC,ppsD
GO:0044550	secondary metabolite biosynthetic process	6	14	0.0194	cysD,cysN,ideR,mbtB,mbtG,mbtI
GO:0046189	phenol-containing compound biosynthetic process	5	7	0.0194	mbtI,ppsA,ppsB,ppsC,ppsD
GO:0071554	cell wall organization or biogenesis	16	95	0.0194	Rv1433,Rv2953,Rv2959c,drrB,fadD23,fadD26,mmpL3,mmpL8,papA5,pks1,ppsA,ppsB,ppsC,ppsD,ppsE,ufaA1
GO:0071840	cellular component organization or biogenesis	24	182	0.0194	Rv1433,Rv2953,Rv2959c,drrB,engA,fadD23,fadD26,ksgA,mmpL3,mmpL8,papA5,pks1,ppsA,ppsB,ppsC,ppsD,ppsE,rimM,rlmN,rnc,rsmE,rsmI,ufaA1,ybeY
GO:0072330	monocarboxylic acid biosynthetic process	16	92	0.0194	bioD,dlaT,fadD23,fadD26,mbtB,mbtI,pks1,pks2,pks4,pks5,ppsA,ppsB,ppsC,ppsD,ppsE,ufaA1
GO:0097040	phthiocerol biosynthetic process	5	7	0.0194	fadD26,ppsA,ppsB,ppsC,ppsD
GO:1901264	carbohydrate derivative transport	4	4	0.0194	drrA,drrB,drrC,mmpL3
GO:0006400	tRNA modification	6	17	0.0321	fmt,rlmN,trmB,trmD,trmI,truA
GO:0097041	phenolic phthiocerol biosynthetic process	4	6	0.0321	ppsA,ppsB,ppsC,ppsD
GO:0008033	tRNA processing	7	25	0.0341	fmt,rlmN,rnc,trmB,trmD,trmI,truA
GO:0042254	ribosome biogenesis	8	33	0.0346	engA,ksgA,rimM,rlmN,rnc,rsmE,rsmI,ybeY
GO:0010106	cellular response to iron ion starvation	5	12	0.0355	Rv0560c,irtA,mbtB,mbtI,pks2

GO:0009712	catechol-containing compound metabolic process	4	7	0.0407	ideR,mbtB,mbtG,mbtI
GO:0019540	siderophore biosynthetic process from catechol	4	7	0.0407	ideR,mbtB,mbtG,mbtI
GO:0042891	antibiotic transport	4	7	0.0407	Rv1634,drrA,drrB,drrC
GO:1901617	organic hydroxy compound biosynthetic process	7	28	0.0453	fadD26,mbtI,ppsA,ppsB,ppsC,ppsD,snoP
GO:0043215	daunorubicin transport	3	3	0.0455	drrA,drrB,drrC
GO:1900753	doxorubicin transport	3	3	0.0455	drrA,drrB,drrC
GO:0010467	gene expression	29	274	0.0468	fmt,gltS,ideR,ksgA,lepA,mmpR5,mtrA,pheT,prrA,rimM,rlmN,rnc,rplS,rplU,rpmA,rpsP,rsbW,rsmE,rsmI,sigH,sigI,sigL,sigM,trmB,trmD,trmI,trpS,truA,ybeY

Table S11. Cellular Component enrichment in the PPI network of the unexpressed proteins of DR strains and other predicted functional partners.

#term ID	Description	Observed Gene Count	Background Gene Count	FDR	Matching Proteins
GO:0032991	protein-containing complex	26	184	0.0085	accD5,atpH,carA,cysD,cysN,dlaT,drrB,drrC,mbtI,obg,pheT,pks4,ppsA,ppsB,ppsC,ppsD,ppsE,rimM,rplS,rplU,rpmA,rpsP,snoP,trmB,trmI,xseB
GO:0034081	polyketide synthase complex	6	10	0.0085	pks4,ppsA,ppsB,ppsC,ppsD,ppsE
GO:1902494	catalytic complex	13	69	0.0145	accD5,carA,cysD,cysN,dlaT,drrB,drrC,mbtI,pheT,snoP,trmB,trmI,xseB
